# AI-Enabled Flexible Sensing Skin for Next-Generation Aircraft: Toward Embodied Intelligence

**DOI:** 10.34133/research.1305

**Published:** 2026-06-08

**Authors:** JingJing Ji, YuXuan Pan, ZhiHan Zhang, Tian Xia, Fan Zhang, YongAn Huang

**Affiliations:** ^1^ State Key Laboratory of Intelligent Manufacturing Equipment and Technology, Huazhong University of Science and Technology, Wuhan 430074, China.; ^2^ Research Center for Advanced Electronics Manufacturing, School of Mechanical Science and Engineering, Huazhong University of Science and Technology, Wuhan 430074, China.; ^3^ Flexible Electronics Research Center, Huazhong University of Science and Technology, Wuhan 430074, China.

## Abstract

Aircraft serve as the intelligent extension of human capabilities into 3-dimensional space. Next-generation aircraft will transcend the limitations of traditional fixed aerodynamic configurations, evolving into embodied intelligent agents capable of sensing their environment, making autonomous decisions, acting collaboratively, and self-evolving—ultimately achieving Fly-by-Feel. Flexible sensing skins, with their unique advantages (ultrathin, lightweight, large-area conformal attachment to the aircraft surface, and adaptive regulation), are emerging as the key enabling technologies. Multi-aerodynamic parameters are acquired through in situ sensing, and artificial intelligence (AI)-enhanced decision-making is implemented via edge-deployed chips. Based on this sensing–algorithm collaborative bridge, closed-loop actuation is further realized to support functional implementation. This paper reviews the core composition of the “sensing–decision–regulation” closed-loop system, including aerodynamic disturbance-free in situ sensing and AI-enhanced super-resolution field perception, and demonstrates its applications in 3 key scenarios: anti-icing/deicing, electromagnetic stealth, and lift enhancement/drag reduction. Finally, it highlights the future prospects of programmable intelligent flexible skins, promoting the aircraft’s paradigm shift toward intelligence, efficiency, and safety.

## Introduction

Future aircraft will evolve into a new generation of embodied intelligent aerial agents, capable of real-time perception of complex flight environments, autonomous decision-making for performance adjustment, and environmental self-adaptation [1,2]. Such evolving intelligence demands that the self-perception system deliver accurate, real-time structural and environmental data to underpin its autonomous learning and decision-making processes. Intelligent flexible sensing skin (iFlexSense) represents a revolutionary technological paradigm. Its core principle lies in integrating multiple flexible sensing and actuation units into an ultrathin substrate film conformally attached to aircraft surfaces, endowing conventional load-bearing structures with integrated functionalities of self-perception, data processing, and autonomous actuation [3]. Unlike conventional micro-electro-mechanical system (MEMS) that focus on single-point measurement, the iFlexSense can be deployed over large areas, even attached to thin-walled regions or internal flow channel areas. Owing to its ultrathin and lightweight nature, it imposes negligible modifications to the original aerodynamic contour when affixed to aircraft surfaces, thereby fully preserving the aircraft’s aerodynamic performance [4,5]. By integrating strain, pressure, shear stress, and other sensors, it acquires critical data regarding the aircraft’s structural state and ambient flow field, which serve as the core operational indicators for aircraft flight [6].

As the core intelligent module, artificial intelligence (AI) furnishes algorithmic support for the precise decision-making of iFlexSense systems [7], on the basis of super-resolution field reconstruction perception. Reconstruction algorithms essentially rely on discrete measurement point data collected by flexible skins, enabling accurate perception of distributed physical field information. Integral calculation of the reconstructed full-surface pressure distribution allows for real-time extraction of the aircraft’s lift and drag, which, in turn, empowers the flight control system to autonomously adjust flight states and realize safer, more energy-efficient flight strategies; precise reconstruction of the ambient flow field enables accurate identification of key flow phenomena including shock waves and boundary layer transition, offering a reliable decision-making basis for the early warning of hazardous flight conditions such as aircraft flutter and stall.

iFlexSense empowers the aircraft to become intelligent agents integrating sensing, perception, and actuation. Closely linked to aircraft flight safety, survivability, and core energy efficiency, anti-icing/deicing [8], electromagnetic stealth [9], and lift enhancement/drag reduction [10] stand as the 3 primary application scenarios for aircraft flexible smart skins. For aircraft anti-icing and deicing, mainstream current approaches predominantly depend on ground preventive protection and in-flight hot-air deicing. Flexible skins integrated with sensing capabilities enable active anti-icing/deicing through hydrophobic surfaces and heating interlayers; when coupled with neural networks (NNs), they deliver predictive support for deicing strategies, markedly boosting deicing efficiency and operational safety. In the domain of electromagnetic stealth, conventional coating-based stealth technologies are constrained by severe frequency-selective limitations. By harnessing electromagnetic metasurfaces and AI-driven adaptive frequency tuning functionalities, the iFlexSense system can realize full-band stealth coverage by regulating the geometric parameters (e.g., array spacing and dimension) of metallic microstructures. In contrast to the fixed aerodynamic configurations of contemporary aircraft, the iFlexSense system permits dynamic control of surface protrusions and even morphing wing structures, thereby optimizing the aircraft’s aerodynamic profile in real time during flight and improving aerodynamic efficiency. Although remarkable progress has been made in this research field, comprehensive and systematic review literature remains scarce.

Adopting the perception–decision–actuation collaborative closed loop as its core framework, this review systematically elaborates on the technological evolution and key breakthroughs of aircraft flexible smart skins in recent years. It further predicts that future flexible smart skins will fully break the design constraints of conventional aircraft that passively conform to aerodynamic environments, driving a paradigm shift toward aircraft that proactively sense airflow conditions and dynamically regulate surface properties. This will provide core impetus for the advancement of aerospace technology toward greater intelligence, efficiency, and safety, establishing it as a revolutionary enabling technology for the development of next-generation intelligent aerial vehicles.

## AI-Enabled iFlexSense System

iFlexSense is an integrated system. Built on flexible substrate materials (e.g., polyimide [PI] films) as its carrier, it integrates sensitive units such as pressure, strain, and temperature sensors onto the substrate via micro-nano fabrication technologies including magnetron sputtering and photolithography, forming a distributed sensing network. Such flexible substrates typically have a thickness of less than 100 μm, while the sensing units are generally fabricated at the submicron scale.

This ultrathin characteristic causes no damage to aircraft structures, nor does it alter their aerodynamic profiles, ensuring exceptional aerodynamic compatibility. However, accurately identifying flight states using limited sensing data remains a considerable challenge due to the strong nonlinearity of the surrounding aerodynamic field. AI-empowered iFlexSense, which integrates AI algorithms with its hardware platform, is the sole technical route capable of simultaneously satisfying the requirements of field perception, rapid decision-making, and adaptive regulation. The key merit of this technology lies in breaking through the constraints of traditional passive sensing, constructing a complete perception–decision–actuation closed loop, and realizing a genuine leap from simple data collection to active intelligent functionality.

### System composition and key technologies

Flexible electronic skins were initially applied in human wearable devices for monitoring physiological signals such as heart rate and body temperature [11]. Subsequently, it found application in industrial robotics for human–machine interaction monitoring [12]. For aircrafts, however, key measurement parameters focus on structural status and aerodynamic parameters. To accurately capture key signals including strain, vibration, and sudden changes in airflow distribution, sensor arrays must achieve micrometer-level spatial resolution and millisecond-level response speed (under high-speed maneuvering conditions, the single-frame response latency must be strictly controlled to ≤10 ms [13], while under flutter conditions, the response latency must meet the requirement of ≤5 ms [14]). To achieve this rapid response requirement, lightweight design is indeed a key technical pathway—among which model quantization and network pruning are the most mainstream lightweight technologies. Specifically, INT8 quantization reduces model volume by 75% [15], and network pruning cuts computational load by 40% to 55% [16], enabling efficient reduction of model size and computational overhead with minimal performance loss. The demanding environmental conditions in aerospace applications impose higher adaptability requirements on flexible skins [17,18]—ensuring no degradation of device performance over a wide temperature range (from low temperatures of −60 to −70 °C at high altitudes [19] to high temperatures of 1,100 °C near engines [20]). Under these thermal gradients and extreme high-altitude ultraviolet irradiation, the environmental adaptability of flexible substrates represents a critical technical challenge. Conventional PI meets general service requirements, yet in ultrahigh-temperature environments, flexible substrates such as microscale mica sheets are typically adopted to prevent brittle fracture and deformation [21]. In harsh high-altitude environments with intense ultraviolet irradiation and high-energy radiation, fluorine/boron-doped PI or PI aerogels can serve as reinforced substrates. The overall complexity during flight dictates the need for large-area measurement. However, achieving high-precision measurements across large areas remains challenging. The core innovation of iFlexSense lies in employing transfer printing technology to batch-transfer high-precision micro-sensor arrays, previously fabricated using techniques such as photolithography, onto large-area aircraft surfaces without damage [22]. To address the challenges of wiring and data busing in large-area high-density sensing arrays, direct printing techniques such as electrohydrodynamic jet printing are employed to integrally fabricate micro-interconnects on flexible skin surfaces (typical line widths: 3 to 5 μm, submicron thickness). Inspired by high-density super-resolution displays [23], a row–column matrix addressing combined with multichannel signal multiplexing architecture is utilized, enabling efficient signal acquisition for M×N-scale (thousands of) sensing nodes with merely M+N micro-interconnects. This design effectively eliminates wiring congestion while retaining the ultrathin and lightweight characteristics of the flexible skin.

Through core technological innovations such as micro-nano transfer and ultrathin fabrication [24,25], flexible skins have successfully addressed the dual challenges of high-precision, large-area measurement and harsh environment adaptation in aerospace scenarios. The massive data collected by these distributed sensor arrays require both millisecond-level local rapid processing during flight to ensure emergency response and in-depth mining and global analysis in ground-based R&D and full-life cycle management to support decision optimization—imposing differentiated adaptation requirements on the computing architecture for data processing. As a localized decision-making unit, edge intelligence represented by field-programmable gate arrays (FPGAs) adopts an end-to-end deployment scheme, and its operating environment constitutes a typical resource-constrained hardware scenario. Accordingly, such intelligent units take embedded lightweight models (e.g., multilayer perceptron [MLP] and convolutional neural network [CNN]) as their core, with a data processing capability of 10^6^ to 10^11^ operations per second, a bandwidth demand ranging from 0.1 kbps to 1 Gbps, and a hardware power consumption of merely 1 mW to 100 W. Based on the above adaptive performance indicators, it can meet the latency requirements of 1 to 100 ms for core real-time scenarios [26,27]. On the other hand, in aircraft ground tests—including scenarios such as wind tunnel testing and full-life cycle health management—complex tasks like aerodynamic iterative optimization, historical flight data analysis, and integrated fleet operation data analysis inevitably generate massive data processing demands. This necessitates cloud intelligence as a supplement to edge intelligence [28]. Cloud intelligence relies on complex AI models such as variational autoencoders (VAEs) and transformers. Its data pattern processing capacity reaches the order of 10^12^ to 10^18^ operations per second, with a latency range of 100 ms to 5 s, typically requiring a bandwidth of 10 Gbps to 10 Tbps, a power consumption of 1 kW per single server, and a total power consumption of MW level for large-scale cloud data centers [29,30]. With its advantages in high computing power and large-scale data processing, it can simultaneously integrate multisource global information, providing a macro decision-making basis for aircraft R&D optimization, maintenance strategy formulation, and overall performance iteration.

**Fig. 1.**
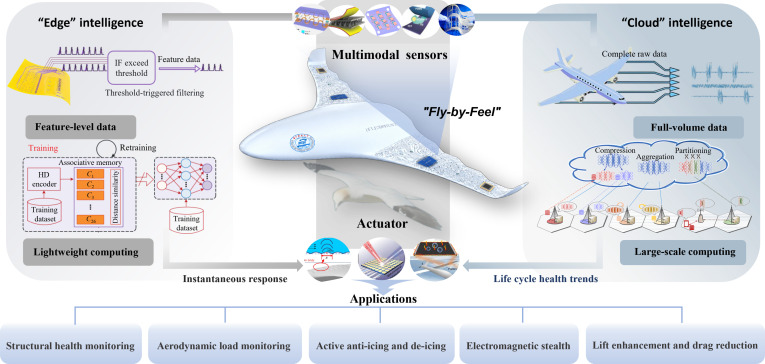
Overview of the artificial intelligence (AI)-enhanced iFlexSense system: Integrating multimodal sensors, intelligent algorithms, and multifunctional actuators to enable structural health and aerodynamic monitoring.

Flexible skins achieve full-domain precise perception through high-precision sensor arrays, while the implementation of core functions such as real-time anti-icing/deicing, electromagnetic stealth, lift enhancement/drag reduction, and structural health monitoring (SHM) requires in-depth integration and adaptation of software and hardware. In the airborne application mode, at the system hardware level, chips such as FPGA suitable for extreme aerospace environments are selected as core processing units. The sensing signal acquisition module and data transmission module of flexible skins are integrated into standardized airborne boards, which transmit collected real-time data (e.g., flow field, strain, and vibration) to the airborne data processing unit or flight control system. Core algorithms—including dynamic ice thickness identification algorithm [31], electromagnetic scattering regulation algorithm [32], structural damage feature extraction algorithm [33], and real-time flow field analysis algorithm [34]—are solidified into the hardware logic units of FPGA chips to realize end-to-end closed-loop processing. In contrast, wind tunnel testing focuses on high-precision aerodynamic detection, long-term structural life cycle monitoring, and design iteration optimization, typically relying on large-scale data acquisition systems and cloud server computing [35]. A multichannel synchronous data acquisition system is built to complete synchronous collection of multidimensional parameters (e.g., structure, airflow, and environment). Data are uploaded to distributed server clusters in real time via dedicated transmission protocols to meet the requirements of massive data storage and high-intensity computing. The software leverages the server’s computing power for the rapid retrieval of historical data and multi-aircraft fleet test data, enabling in-depth comprehensive analysis. Additionally, a digital visualization interface is created to support retrospective comparison and horizontal analysis, facilitating full-life cycle SHM and assisting in the iterative optimization of aircraft design [36]. At this point, a complete AI-enhanced iFlexSense system architecture takes shape (Fig. 1).

### iFlexSense development milestones

**Fig. 2.**
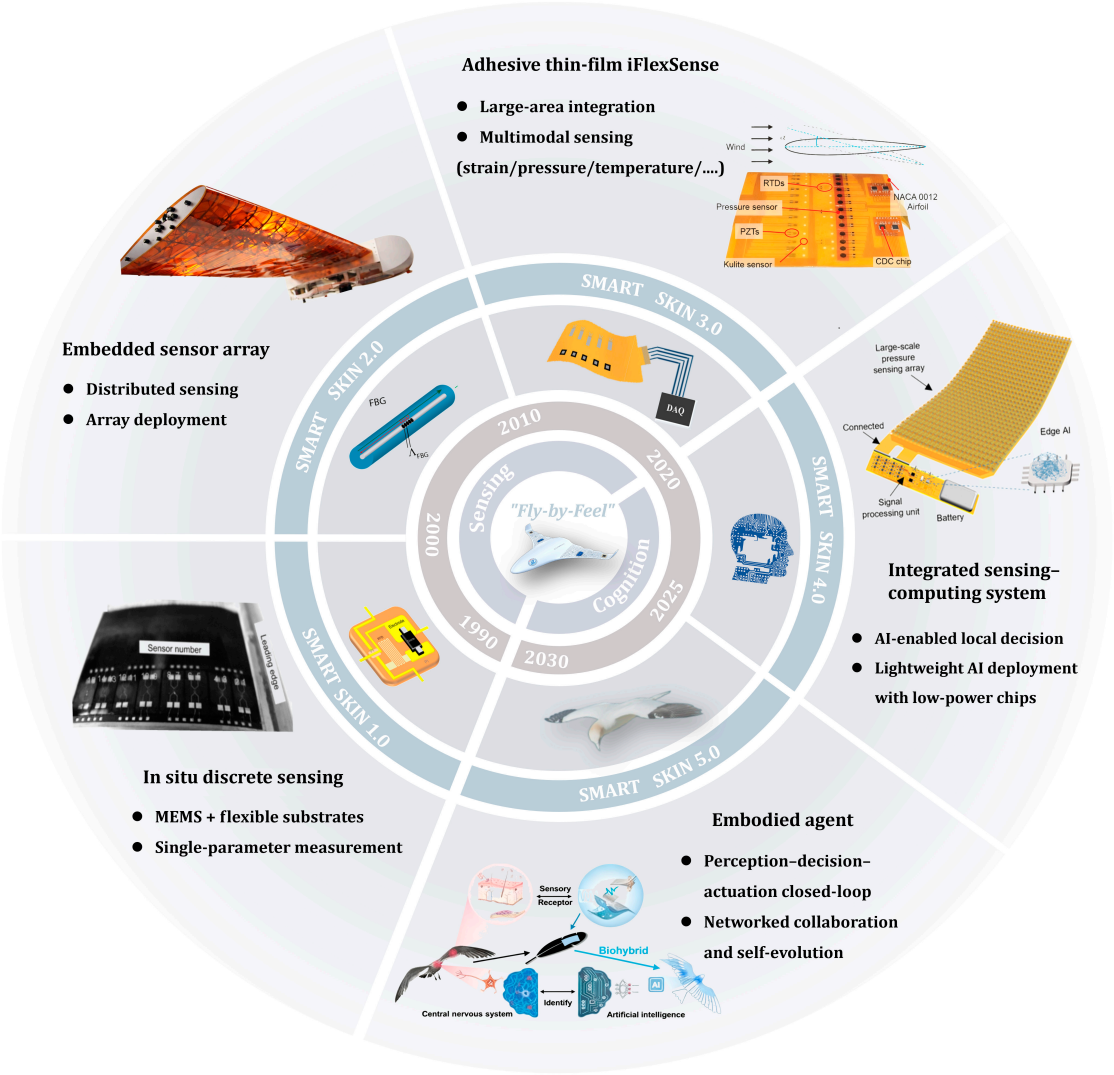
The chronological evolution of aircraft smart flexible skin, highlighting representative AI-enabled technological advancements. From basic sensing units to integrated intelligent systems, the development of aircraft smart flexible skin is categorized into 5 stages—Smart Skin 1.0 to 5.0. Stages 1.0 to 3.0 focus on iterative upgrades of sensing capabilities, achieving gradual expansion in sensing targets, parameter dimensions, and integration levels. In Stage 4.0, deep integration of AI begins, marking a paradigm shift from “sensing” to “perception” in system functionality. Stage 5.0 further evolves toward embodied intelligent systems integrating the closed-loop control of “perception–decision–action”.

The concept of “intelligent skin” was first proposed by the U.S. Air Force in 1985. Its original vision was to directly integrate functional modules into the airframe skin, thereby enabling the aircraft surface to serve as part of a distributed sensing and electronic warfare system [37]. The evolution from basic perception to intelligent skin systems can be broadly understood as a progressive transition from sensing to cognitive capability, divided into 5 stages (Fig. 2). Among them, Stages 1.0 to 3.0 mainly reflect the continuous evolution of sensing, progressing from in situ discrete sensing in Stage 1.0, to embedded sensor arrays in Stage 2.0, and finally to large-area integration and multimodal sensing in Stage 3.0, giving rise to adhesive thin-film iFlexSense. Stage 4.0 marks the extension of iFlexSense toward cognitive, where AI-driven technologies enable iFlexSense to evolve into an integrated sensing–computing system for local decision-making by near-sensor and in-sensor intelligence. Ultimately, in Stage 5.0, intelligent skin is envisioned to evolve into an embodied intelligent agent with a closed-loop perception–decision–actuation architecture, thereby realizing the paradigm of Fly-by-Feel.

Stage 1.0 was characterized by in situ sensing based on MEMS devices. In the 1990s, MEMS began to be integrated onto flexible substrates such as PI to measure surface shear stress, shock motion, and flow separation. The first flexible MEMS skin, developed at Caltech, adopted a silicon-island/PI interconnect architecture, enabling curved-surface attachment and dense sensor integration [38]. Subsequent work improved compatibility with microfabrication processes and enabled 3-dimensional (3D) transient shear-stress measurements in wind-tunnel environments [39]. NASA flight tests further demonstrated that PI-based hot-film arrays could capture shock-position variations during flight, while hybrid AeroMEMS arrays were capable of identifying near-wall separated flow [40]. Although this stage enabled conformal sensing on high-curvature surfaces, it remained limited by small coverage areas, insufficient interfacial reliability, and poor conformability to complex surfaces, allowing only single-parameter measurements.

Stage 2.0 marked the transition of sensing technology from localized measurement points to embedded sensor arrays along structural pathways, achieving distributed sensing. The representative technology of this stage was the fiber Bragg grating (FBG) sensor. Owing to its advantages of high sensitivity, immunity to electromagnetic interference, light weight, and multiplexed networking capability, FBG was widely applied in aerospace SHM and flight testing. By being embedded within composite structures, it enabled the formation of meter-scale sensing networks over wings, fuselages, and other components [41,42]. By integrating multiple gratings within a single optical fiber, multipoint strain and temperature measurements could be achieved [43]. This stage substantially expanded the sensing range and improved signal stability, allowing intelligent skin to further evolve into a distributed monitoring network linking aerodynamic shape with the state of load-bearing structures.

With advances in flexible electronics and materials science, Stage 3.0 achieved large-area integration and multimodal sensing, giving rise to adhesive thin-film iFlexSense meeting the demands for full-surface, multiparameter, and high-resolution measurements over complex aerodynamic structures. The introduction of novel functional materials, including conductive polymers, liquid metals, and 2-dimensional (2D) materials, endowed sensors with bendability, stretchability, and self-healing capability while maintaining excellent electrical performance [44,45]. Meanwhile, structural designs based on serpentine or fractal interconnects, stretchable conductive composite networks, and programmable array layouts tightly integrated sensors, signal acquisition circuits, and flexible printed circuit boards into networked topologies. These not only ensured the local independence of individual sensing nodes but also enabled global information fusion, thereby supporting real-time aerodynamic state monitoring and structural health assessment [46,47]. Under wind-tunnel and flight conditions, such flexible electronic skins enabled high-resolution pressure and strain measurements on unmanned aerial vehicle (UAV) wings or other complex surfaces, and could reconstruct the global aerodynamic field through networked nodes, thus realizing the transition from single-point measurement to full-surface, multiparameter integrated sensing [48].

Entering the 2020s, as full-surface and multiparameter sensing architectures gradually matured, intelligent skin systems were increasingly required to extract flight-relevant key information from massive heterogeneous sensor data in real time. Constrained by bandwidth, latency, and power consumption, traditional approaches relying on external centralized processing became increasingly inadequate. This drove iFlexSense beyond enhanced sensing toward an AI-enabled stage of cognitive enhancement, namely, Stage 4.0, becoming an integrated sensing–computing system. At this stage, machine learning can be used to identify critical states such as flow separation, stall, and flutter [3], and can also reconstruct full-field pressure distributions from limited sensing points [49]. The coupling of AI and materials further shortened the conventional chain of “sensing–sampling–transmission–computation”. In this context, the 2 complementary pathways of intelligent metamaterials and metamaterials intelligence, corresponding respectively to AI for materials and materials for AI, embed part of the sensing and computing burden directly into the material and structural design itself [50]. Through such lightweight deployment, intelligent skin acquires perception–decision capability and evolves from a passive data acquisition unit into a distributed information node capable of anomaly identification, state estimation, and local decision support.

With continued advances in flexible multimodal sensing, embedded AI, and miniaturized actuation, intelligent skin is expected to evolve into Stage 5.0—an embodied intelligent agent. In this stage, materials, structures, sensing, computing, and actuation converge into a closed-loop “perception–decision–actuation” system. Under the Fly-by-Feel paradigm, the aircraft surface transforms from a passive sensing interface into an active participant in flight perception and adaptive regulation [51]. Existing studies have combined feather-piezoelectric mechanoreceptors and deep learning to achieve real-time perception of flight parameters [52], allowing AI to perceive physical fields through material responses. For sensor hardware design, AI can enhance the sensitivity, conformability, and environmental adaptability of flexible skin by optimizing the composition and proportion of tunable materials and regulating surface microstructural units [53]. For intelligent information processing, lightweight in situ deployment is also a critical step to realize the deep integration of AI at the material level and establish a fully embodied intelligent agent. Lightweight AI algorithms are directly deployed on flexible sensing hardware through model compression, hyperdimensional computing, and other technologies, enabling flexible electronic skin to act as an embodied intelligent terminal with real-time local intelligent response [54]. Ultimately, intelligent skin will become an embodied intelligent node featuring endogenous physical intelligence, networked collaboration, and continuous evolutionary potential [55].

## Aerodynamic Multiparameter In Situ Sensing

The sensing requirements for aircraft flexible skins closely revolve around 2 core objectives: flight safety assurance and aerodynamic performance optimization. As the skin interacts directly with airflow, it is necessary to real-time acquire the aerodynamic loads generated by the surrounding flow field—including the normal pressure (which affects lift) and the shear force (which is associated with drag and control stability). In terms of structural safety, the skin endures airflow impact, vibration fatigue, and temperature changes over the long term, making it prone to micro-crack initiation, propagation, or material performance degradation. Therefore, it is essential to accurately capture parameters such as the structural strain distribution, vibration frequency, and amplitude. To meet these measurement requirements, functional sensors are deployed in a targeted manner: pressure sensors are used to obtain normal aerodynamic pressure for lift inversion; shear force sensors directly detect the tangential force of airflow; strain sensors monitor structural deformation; and vibration sensors capture dynamic mechanical responses. By integrating micro-sensor arrays with excellent independent detection capabilities onto the flexible skin substrate, and relying on the collaborative arrangement of multitype sensing units, the flexible sensing skin enables simultaneous detection of multiple physical quantities.

### Aircraft lift determination–aircraft lift–normal pressure

**Fig. 3.**
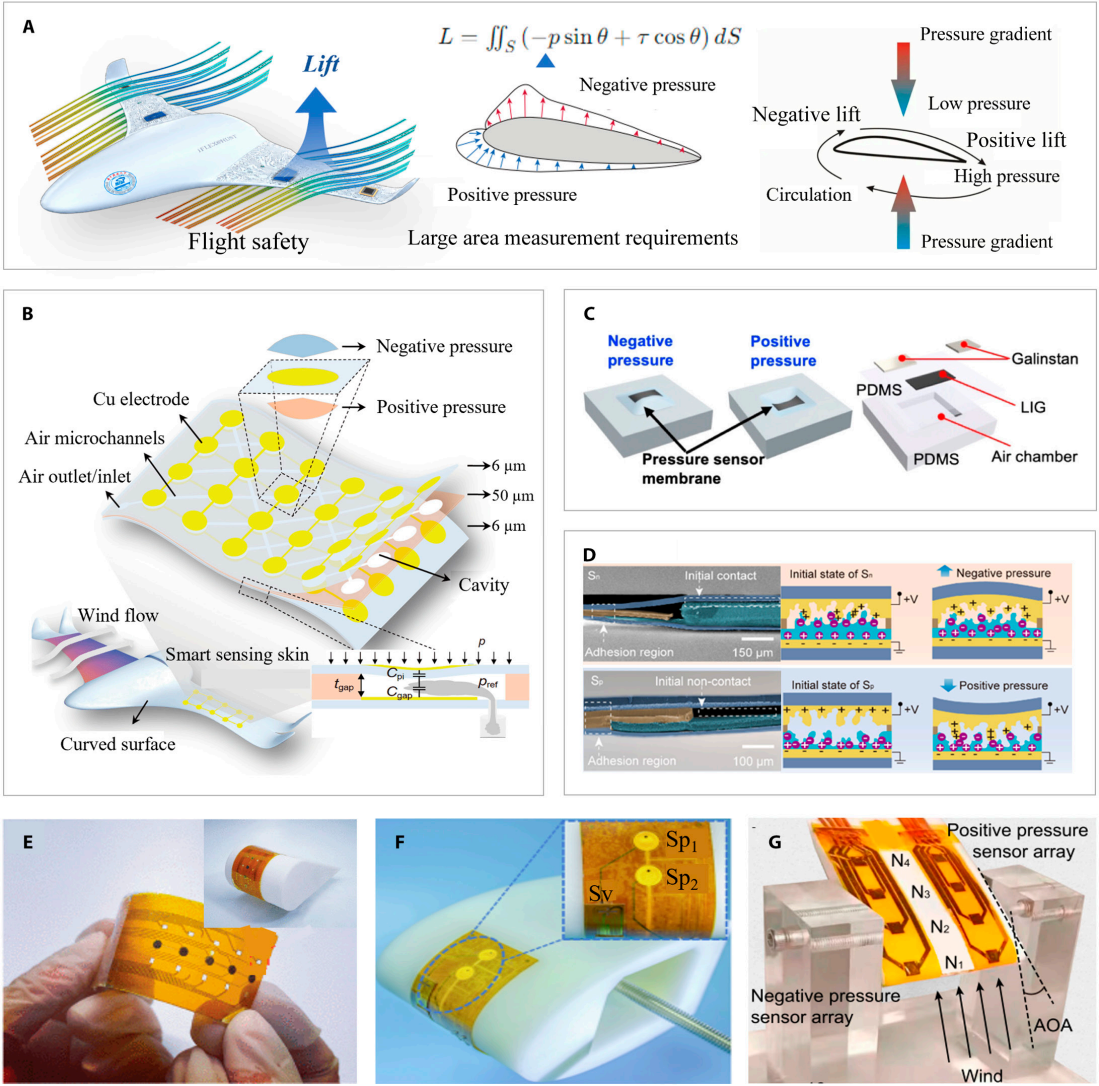
Measurement of aircraft lift—pressure sensor (A) Schematic diagram of an aircraft equipped with a flexible skin for wind pressure measurement, and the pressure distribution across a wing surface. *P* and *U* represent the wing surface pressure and the wind velocity, respectively. (B) Schematic diagram of the future UAV equipped with smart sensing skin for feeling wind pressure and principle of tuning the sensing ranges. Reproduced with permission from Ref. [48]. Copyright 2020, Springer Nature. (C) Schematic images of the flexible air pressure sensor at negative/positive pressure and the structure of the air pressure sensor. Reproduced with permission from Ref. [60]. Copyright 2024, American Chemical Society. (D) The cross-section of *S*_n_ and *S*_p_ shown by SEM images and the sensing mechanism of iontronic sensors for negative and positive pressure measurement. Reproduced with permission from Ref. [61]. Copyright 2024, The Author(s). (E) Photograph of the flexible skin with pressure and airflow sensor arrays, and the inset is the flexible skin attached to an airfoil model. Reproduced with permission from Ref. [64]. Copyright 2023, IEEE. (F) The flexible sensing patch mounted on the wing model. Reproduced with permission from Ref. [70]. Copyright 2025, The Author(s). (G) Standard NACA0012 airfoil integrated with ionic gel-based capacitive pressure sensor array for wind pressure measurement in wind tunnel experiments. Reproduced with permission from Ref. [61]. Copyright 2024, Springer Nature.

The measurement of pressure distribution on the aircraft wing surface is the basis for obtaining lift, which is the core factor for ensuring flight safety and optimizing aerodynamic performance. According to Bernoulli’s principle, lift is essentially the cumulative effect of the pressure difference between the upper and lower surfaces of the wing [56], and it can be obtained by integrating the pressure over the wing surface (Fig. 3A)—this is also the fundamental reason why large-area pressure measurement is required. Furthermore, abrupt pressure changes are also “direct signals” for capturing key aerodynamic phenomena such as shock wave positions and flow separation, which is highly related with stall risk [57]. Measuring the surface pressure of an aircraft wing requires balancing multiple metrics: the sensitivity must be sufficient to capture subtle pressure changes for identifying airflow anomalies; the measurement range should cover the positive–negative pressure difference range while ensuring rapid dynamic response to track transient pressure; additionally, it must meet the requirements of environmental stability and miniaturized installation.

Compared with traditional sensing technologies such as pressure scanning valves [58], MEMS chips [59], and fiber optic sensors, the ultrathin flexible intelligent skin with the advantages of surface fit and aerodynamic compatibility shows great application prospects, avoiding the interference of sensor installation on the airflow field from the root, and adapting to the limited installation space on the wing surface [48] (Fig. 3B). The core sensing mechanisms of pressure sensors are mainly divided into 2 categories: resistive and capacitive sensing. Resistive sensors are based on the piezoresistive effect. Pressure-induced deformation of the sensitive layer changes the connectivity of its internal conductive paths, thereby causing a substantial change in resistance [60] (Fig. 3C). Such sensors have simple manufacturing process and low cost. Capacitive sensors, on the other hand, center on a capacitive structure composed of flexible electrodes and a dielectric layer. External wind pressure causes telescopic deformation of the dielectric layer, leading to regular fluctuations in capacitance [61] (Fig. 3D). Their advantages include fast response speed and excellent temperature stability, making them suitable for real-time monitoring of dynamic aerodynamic pressure and thus more favored in wing surface pressure measurement.

Research on substrate membranes and sensitive cell materials constitutes a pathway toward achieving superior sensor performance [62,63]. PI has been widely used due to its excellent mechanical properties and thickness-tunable fabrication techniques [64] (Fig. 3E). To address the measurement of both positive and negative pressures, the cavity structure has emerged as an effective solution. The cavity structure allows pressure differences to induce changes in the distance between 2 electrodes, thereby leading to capacitance variations [65]. Breakthroughs in precision machining processes such as laser etching and lithography have promoted the rapid development of higher-density, finer-structured sensor array fabrication technologies [66]. Xiong et al. [48] pioneered a programmable capacitive pressure sensor based on a PI substrate and a microcavity structure, with a thickness of only 70 μm and a high sensitivity of 0.28 kPa^−1^ within the range of 0 to 3 kPa. To enhance sensitivity, researchers have fabricated sensors by combining flexible substrates with abundant surface functional groups and conductive materials [60]. Notably, improving the dielectric constant is also a crucial means to boost the sensitivity of capacitive sensors. By integrating graphene fibers into fabrics, Javed et al. [67] developed sensors with layered conductivity, substantially enhancing sensitivity. With the advent of the electric double-layer principle, ionic liquid-based capacitive sensors have garnered widespread attention from researchers [68,69]. These sensors can not only detect subtle changes in the pressure layer induced by airflow separation via a high-sensitivity sensing chip, but also cover both positive and negative pressure ranges. Wang et al. [61] reported an ionic gel-based capacitive pressure sensor with an ultrawide sensing range (–100 to 600 kPa) and a high-frequency response of 400 Hz (Fig. 3G). Nonnegligible interference from temperature variations affects the testing performance of most industrial wind tunnels. To solve this issue, Li et al. [70] proposed a differential cavity structure, which eliminates crosstalk caused by temperature changes (Fig. 3F). The performance metrics of flexible skins under normal pressure are summarized in Table 1.

**Table 1.** Performance metrics of flexible skins under normal pressure

Index	Sensitivity	Measurement range	Response time	Temperature stability	References
1	10.2% kPa^−1^ (Positive pressure) −0.55% kPa^−1^ (Negative pressure)	−30–30 kPa	30–40 ms	20–60 °C	[60]
2	1.60 × 10^3^ pF·kPa^−1^ @ −10 to 0 kPa 1.69 × 10^4^ pF·kPa^−1^ @ −20 to −10 kPa 1.58 × 10^3^ pF·kPa^−1^ @ −20 to −100 kPa	−100–600 kPa	2.5 ms	25–90 °C	[61]
3	3.1 fF·MPa^−1^ @ 0–12 MPa 5.3 fF·MPa^−1^ @ 4–20 MPa	0–20 MPa	–	RT	[65]
4	2.0 × 10^2^ fF·kPa^−1^	−1.0–1.0 kPa	ms level	RT	[70]

At present, the key challenge to be addressed for capacitive pressure sensors is the sampling frequency to cope with high-frequency pulsating signals. Future flexible skins will develop toward the direction of “larger size, thinner thickness, and higher precision”, enabling the detection of airflow separation during high-angle-of-attack flight.

### Aircraft drag determination—shear stress

**Fig. 4.**
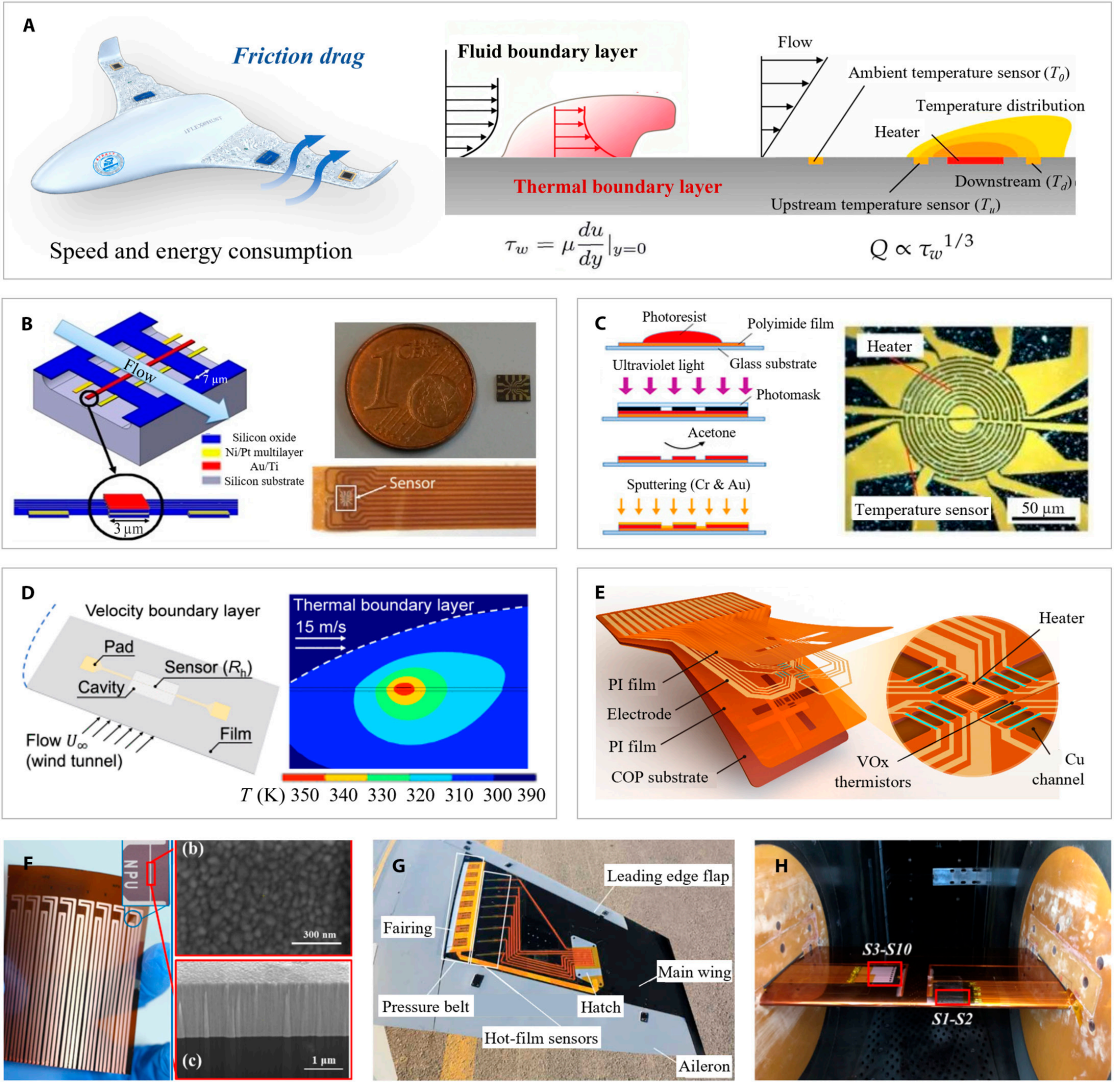
Measurement of aircraft shear force—friction sensor. (A) Schematic diagram of boundary layer velocity and shear stress measurement principle, and the principle of the thermal flow sensor for wall shear stress and flow angle. (B) Calorimetric sensor for bidirectional wall shear stress measurement: sensor chip scaled with euro cent and flexible packaging. Reproduced with permission from Ref. [77]. Copyright 2017, Elsevier. (C) Fabrication process of the flexible flow sensor; picture of heater and temperature sensor in the MEMS flow sensor and the yellow area is polyimide substrate, and the black area is Au thin film. Reproduced with permission from Ref. [78]. Copyright 2022, The Author(s). (D) Schematic of the suspended FSS sensor. When fluid flow *U*_∞_ occurs, a velocity boundary layer is formed on the sensor surface, with the generated shear forces *τ* and associated heat transfer. Reproduced with permission from Ref. [79]. Copyright 2024, IEEE. (E) Exploded view of a thin flexible calorimetric flow (FCF) sensor with high sensitivity and directionality. Reproduced with permission from Ref. [80]. Copyright 2024, The Author(s). (F) Photograph of the flexible hot-film sensor and SEM microscopy images of the Ni thermistor film (surface and cross-section). Reproduced with permission from Ref. [83]. Copyright 2020, IOP Publishing. (G) Experimental photograph of the right wing attached hot-film sensors. Reproduced with permission from Ref. [81]. Copyright 2023, IOP Publishing. (H) Experimental setup of flexible hot-film sensors on the airfoil surface in the NF-6 high-speed continuous transonic wind tunnel. Reproduced with permission from Ref. [83]. Copyright 2020, IOP Publishing.

Normal pressure is directly related to aircraft lift, while tangential shear stress directly reflects the magnitude of wall friction drag on the wing surface, which is another core object of aircraft monitoring. The fundamental cause of aircraft wall friction drag is the interaction between air viscosity and the aircraft surface [71]. This adhesion effect makes the boundary layer form an obvious velocity layer, and the viscous shear between air molecules, air flow, and the fuselage surface produces shear stress (Fig. 4A). According to Kolmogorov’s turbulent statistical theory [72], in the aerodynamic turbulent boundary layer, the size of the smallest vortices is approximately 10 to 100 μm, and the formation and dissipation time of these small vortices is only 0.1 to 1 ms. It is enough to prove that the shear stress has the characteristics of small numerical value, high dynamics and small characteristic scale of the flow structure. Therefore, in recent years, the wall shear stress sensor has been improved in terms of ultrathinning, high sensitivity, and high reliability.

The wall shear stress measurement technology mainly uses the traditional floating MEMS shear stress sensor [73], oil film method [74], hot wire anemometer [75], etc. As a modern technology for wall shear stress measurement, the flexible hot-film sensor is essentially a flexible electronic sensing thin-film element that works based on the self-heating effect. It first attains a stable thermal state via self-heating, then establishes the correlation between wall shear stress and its temperature through forced convective heat transfer [76]. Featuring a typical thickness of less than 100 μm, the flexible thermal sensor is suitable for direct attachment to aircraft wings, enabling wall shear stress measurement [77] and adaptive performance characterization in extreme operating environments (e.g., subsonic flow fields) [78] (Fig. 4B and C).

Research in the field of flexible hot-film sensors in aerospace primarily focuses on the development of high-performance flexible substrate materials and the optimization of micro-nano structures. Song et al. [79] prepared a suspended ultrathin flexible hot-film sensor with a thickness of only 6 μm, a sensitivity of 1.99 V^2^/Pa^1/3^, and a fast response characteristic of 1.2 ms (Fig. 4D). Also focusing on suspended structures, Gong et al. [80] designed a flexible calorimetric flow sensor based on a high-sensitivity vanadium oxide thermistor with an unprecedented speed resolution of 0.11 mm/s (Fig. 4E). Pang et al. [81] developed a 2-layer hot-film sensor, which improved its sensitivity by approximately 150% through reducing heat conduction (Fig. 4G). To meet the multiparameter measurement requirements of complex scenarios, they further developed an integrated sensor for shear stress, flow direction, and dynamic pressure on this basis [82]. This integrated sensor can measure wall shear stress in the range of 0 to 17 Pa with a measurement error of less than 0.4 Pa and a directional error of less than 3°. In addition to structural optimization, the regulation of the key performance of the sensor by the fabrication process is also crucial (Fig. 4F). Sun et al. [83] investigated the effects of annealing temperature and duration on the temperature coefficient of resistance (TCR), fabricated a flexible hot-film sensor with a TCR of 5,100 ppm/°C, and further studied the flow transition and separation characteristics of the NACA 0012 airfoil under various operating conditions (Fig. 4H). The performance metrics of flexible skins under shear stress are summarized in Table 2.

**Table 2.** Performance metrics of flexible skins under shear stress

Index	Sensitivity	Measurement range	Response time	Temperature stability	References
1	CC mode: resistance variation 0.53% CT mode: current variation > 1%	0–2.4 Pa	500 μs	20–70 °C	[76]
2	1.99 V^2^·Pa^−1/3^	0–2.5 Pa	1.2 ms	25–50 °C	[79]
3	2.43 V^2^·Pa^−1/3^	0.3–2.6 Pa	ms level	10–65 °C	[81]

The flexible hot-film sensor is developing rapidly, which has the characteristics of good surface conformality, high sensitivity, and fast response time. However, heat loss control and dynamic calibration remain 2 major challenges that need to be addressed. Future development will continue to focus on material innovation, structural optimization, and other directions, gradually realizing the leap from laboratory research to engineering applications.

### Aircraft structural monitoring—strain and vibration

**Fig. 5.**
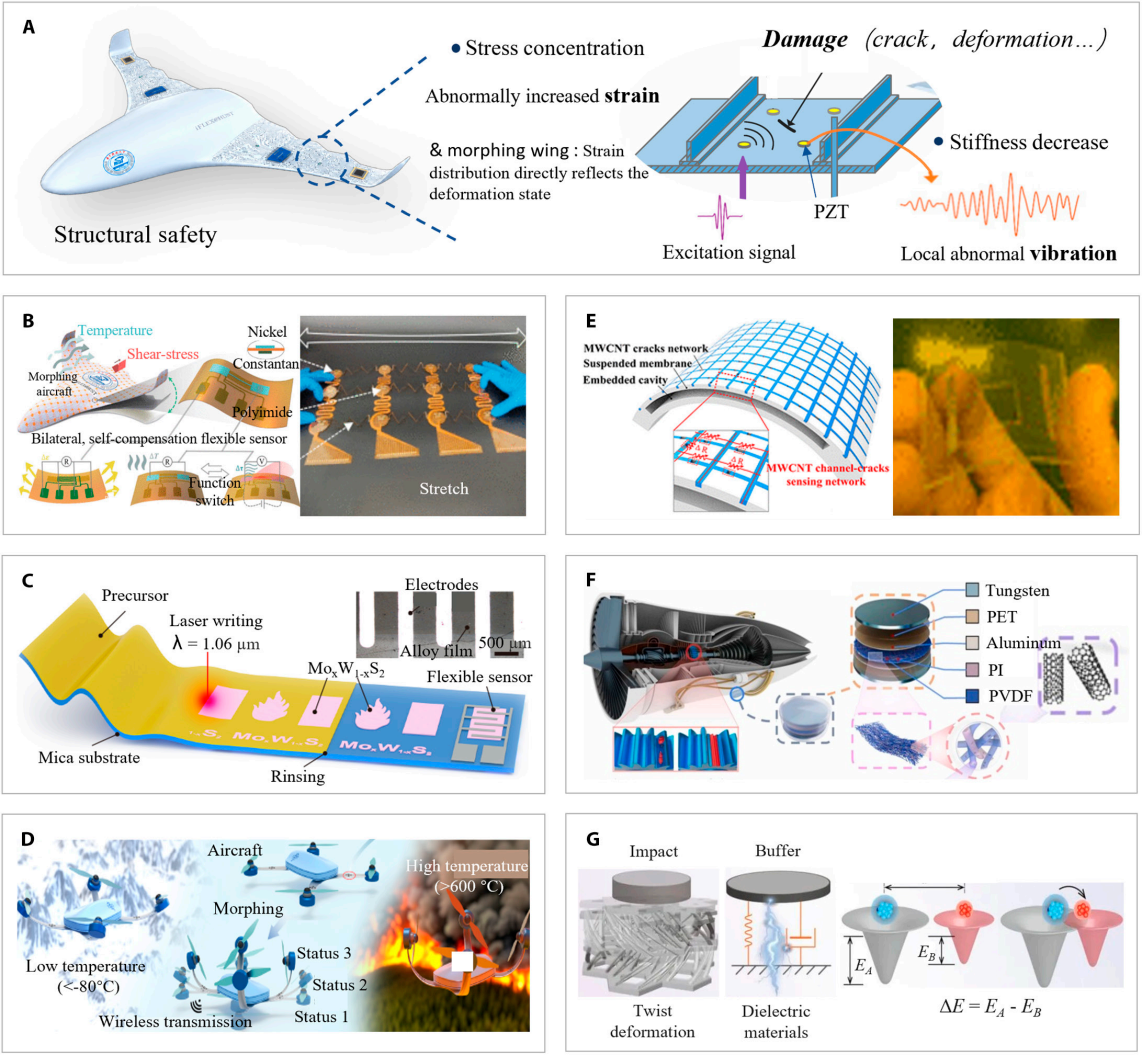
Monitoring of aircraft structural monitoring—strain and vibration sensors. (A) Typical aircraft structural damage. The 4 diagrams corresponding to the lower left part are the strain sensors: (B) application scenario, physical modeling, working principle, and compensation mechanism of the bilateral flexible sensor with integrated in situ self-compensation; photographic image of the smart skin with island-bridge structure under stretched state. Reproduced with permission from Ref. [92]. Copyright 2025, IEEE; (C) fabrication of MoxW1-xS2-based flexible sensors. Inset shows an optical image of the alloy film and electrode. Reproduced with permission from Ref. [95]. Copyright 2025, The Author(s); (D) 4 flexible painting sensors are attached to the wings for monitoring the flying and morphing status of the aircraft at various extreme conditions with different temperature regimes. Reproduced with permission from Ref. [21]. Copyright 2025, The Author(s). The 4 diagrams corresponding to the lower right part are strain sensors: (E) schematic diagram of the flexible vibration sensor and the photograph of the flexible vibration sensor. Reproduced with permission from Ref. [97]. Copyright 2021, American Chemical Society; (F) structure diagram and application schematic of the PVDF-PI nanofiber membrane-based piezoelectric vibration sensor for aircraft engine gear fault monitoring. Reproduced with permission from Ref. [98]. Copyright 2025, Elsevier; (G) working principle of the THM-TENG for self-powered sensing and impact buffering: mechanical-to-electrical energy conversion during impact. Reproduced with permission from Ref. [99]. Copyright 2025, The Author(s).

Aircrafts inevitably face deformation, fatigue, corrosion, and other losses during long-term flight, and monitoring the structural safety of the aircraft itself is a “bottom line requirement” for aviation safety. If structural hazards such as stress concentration and stiffness reduction occur, catastrophic accidents such as wing breakage and fuselage disintegration may occur in severe cases. Strain sensing and vibration sensing are key monitoring tools for identifying health hazards in aircraft structures [84]. Strain refers to the micro-deformation of a structure after being loaded, and it has a linear relationship with stress. Strain sensors can directly and quantitatively reflect the stress state of the structure—determining the presence of stress concentrations or predicting the initiation of fatigue cracks [85]. Structural stiffness directly dictates natural vibration characteristics. By tracking the dynamic characteristics of the loaded structure in real time, vibration sensors can rapidly detect stiffness degradation signals [86]. These 2 technologies convert invisible structural problems into quantifiable monitoring data, thereby enabling early warning of potential hazards. As more advanced morphing wing technologies continue to develop [87], the dynamic deformation characteristics of structures will become more complex. Flexible sensors that can fit complex structures greatly enhance the comprehensiveness and adaptability of monitoring (Fig. 5A).

The identification of stress concentration relies on tiny strain differences, which imposes strict demands on the sensor’s thickness [88], accuracy [89], and sensitivity [90]. Various research and breakthroughs were conducted, as shown in Fig. 5 (lower left). Yu et al. [91] proposed a laser-induced interface ablation method to fabricate ultrathin graphene-polyimide strain sensors, which measure only 8 μm thick—6 times thinner than those fabricated via traditional surface ablation methods. Guo et al. [92] developed a double-sided flexible sensing skin integrated with in situ self-compensation. The sensor achieves a strain linearity of 99.98%, making it suitable for multimodal measurement of morphing aircraft wings (Fig. 5B). The S^4^ method proposed by Meng et al. [93] obviates the trade-off between spatial resolution and strain resolution, allowing for more accurate detection of regions with steep strain gradients, such as edges and sharp corners. When an aircraft operates from ground level to high altitudes and from tropical to frigid regions, strain sensors must additionally exhibit full environmental tolerance [94]. Wang et al. [95] fabricated Mo*x*W_1−*x*_S_2_ alloy flexible strain sensors via direct pulsed laser. These sensors have a detection limit as low as 4.9 με and can operate stably at a high temperature of 500 °C (Fig. 5C). In contrast, the flexible strain sensor based on a MoWNb medium-entropy alloy coating, developed by Li’s et al. [21], boasts an even lower detection limit of 0.57 με and operates over a wide temperature range from −150 to 1,100 °C (Fig. 5D). The performance metrics of flexible skins under strain are summarized in Table 3.

**Table 3.** Performance metrics of flexible skins under strain

Index	Sensitivity	Measurement range	Response time	Temperature stability	References
1	Gauge factor: GF = 24.8	0–2% @ linear range 3.2% @ fracture strain	50 ms	–	[91]
2	GF = 97.4 @ RT GF = 87.8 @ 300 °C GF = 40.1 @ 500 °C	−392–392 με	0.45–0.46 s @ 20–200 °C; 0.73 s @ 500 °C	20–500 °C	[95]
3	GF = 102.6 @ 0%–1% strain GF = 249.5 @ 1%–3% strain GF =593.3 @ 3%–5% strain	0.57–4,541 με	34 ms	−150–1,100 °C	[21]

In response to the needs of aircraft structural vibration measurement, several research teams have also made innovative attempts, as shown in Fig. 5 (lower right). Early structural hazards only induce subtle vibration signal changes. Sensors require high sensitivity and low noise to distinguish normal fluctuations from early anomalies. Langat et al. [96] adopted the lamination and curing molding process to fully embed piezoelectric sensors into composite laminates, which can effectively avoid the masking of damage characteristics by environmental noise and obtain structural monitoring data with distinct dynamic features. Chen et al. [97] created a suspended-film flexible vibration sensor based on channel crack design. It demonstrates high sensitivity, good reproducibility, and robust sensing stability, and can achieve an ultrawide frequency vibration response range of 0.1 to 20,000 Hz (Fig. 5E). In some special parts of aircraft (e.g., high-temperature areas like engines), sensors are required to have high-temperature stability [20]. Hu et al. [98] proposed a self-powered piezoelectric vibration sensor based on PVDF-PI nanofiber membrane, which only suffers 15% performance loss at 80 °C (Fig. 5F). It is worth mentioning that in terms of functional integration, Chao et al. [99] achieved performance upgrades through the integration of biomimetic structures and metamaterials, and designed a self-powered biomimetic twisted hyperbolic metamaterial-integrated triboelectric nanogenerator sensor [100]. Weighing merely 10 g and with a 36-ms response time, it can perform self-powered monitoring of impacts within 1,000 N and UAV localization, achieving a balance of light weight, multifunctionality, and self-sustainability (Fig. 5G). The performance metrics of flexible skins under vibration are summarized in Table 4.

**Table 4.** Performance metrics of flexible skins under vibration

Index	Sensitivity	Measurement range	Response time	Temperature stability	References
1	GF = 102.6 @ 0%–1% strain GF = 249.5 @ 1%–3% strain GF =593.3 @ 3%–5% strain	0.24–100 m·s^−2^ 0.1–20,000 Hz	40 ms	–	[97]
2	1.95 V·0.3 μA^−1^ @ ~26 Hz	5–25 m·s^−2^ <1,000 Hz	ms level	20–80 °C	[98]
3	~0.24 V·N^−1^ @ 2 Hz	50–1,000 N (impact force) ≤5 Hz	36 ms	−20–60 °C	[99]

As the aviation industry demands more refined and intelligent structural safety monitoring, large-scale application of flexible vibration and strain sensors in aircraft has become inevitable. This will provide core technical support for full-life cycle aircraft health management.

## AI Algorithm-Enhanced Intelligent Perception

The Fly-by-Feel flexible intelligent aircraft relies on flexible sensors to achieve proximal sensing of its flight status. However, sensor hardware alone cannot directly identify the structural health status or flow field evolution laws behind the data. AI algorithms are required to enhance perception capabilities and realize the conversion of “data–information–decision”, so that the flexible intelligent skin is not just “sensing” but “perception”. This section will elaborate on the discussion from 2 aspects: the structure itself and the surrounding aerodynamic field. Regarding fatigue damage and crack propagation induced by the complex operating conditions of the aircraft structure during long-term operation, AI technology can filter out noise, identify early-stage weak damage features, and predict damage evolution trends to generate maintenance decisions. On the other hand, in the study of the aircraft surrounding flow field, flow field reconstruction technology is crucial for obtaining a complete flow field state—by integrating advanced algorithms, it can inversely calculate the full-field flow distribution from discrete iFlexSense measurement points, providing a solid basis for optimizing aircraft aerodynamic performance and controlling flight attitudes.

### Structural health self-monitoring and self-diagnosis

**Fig. 6.**
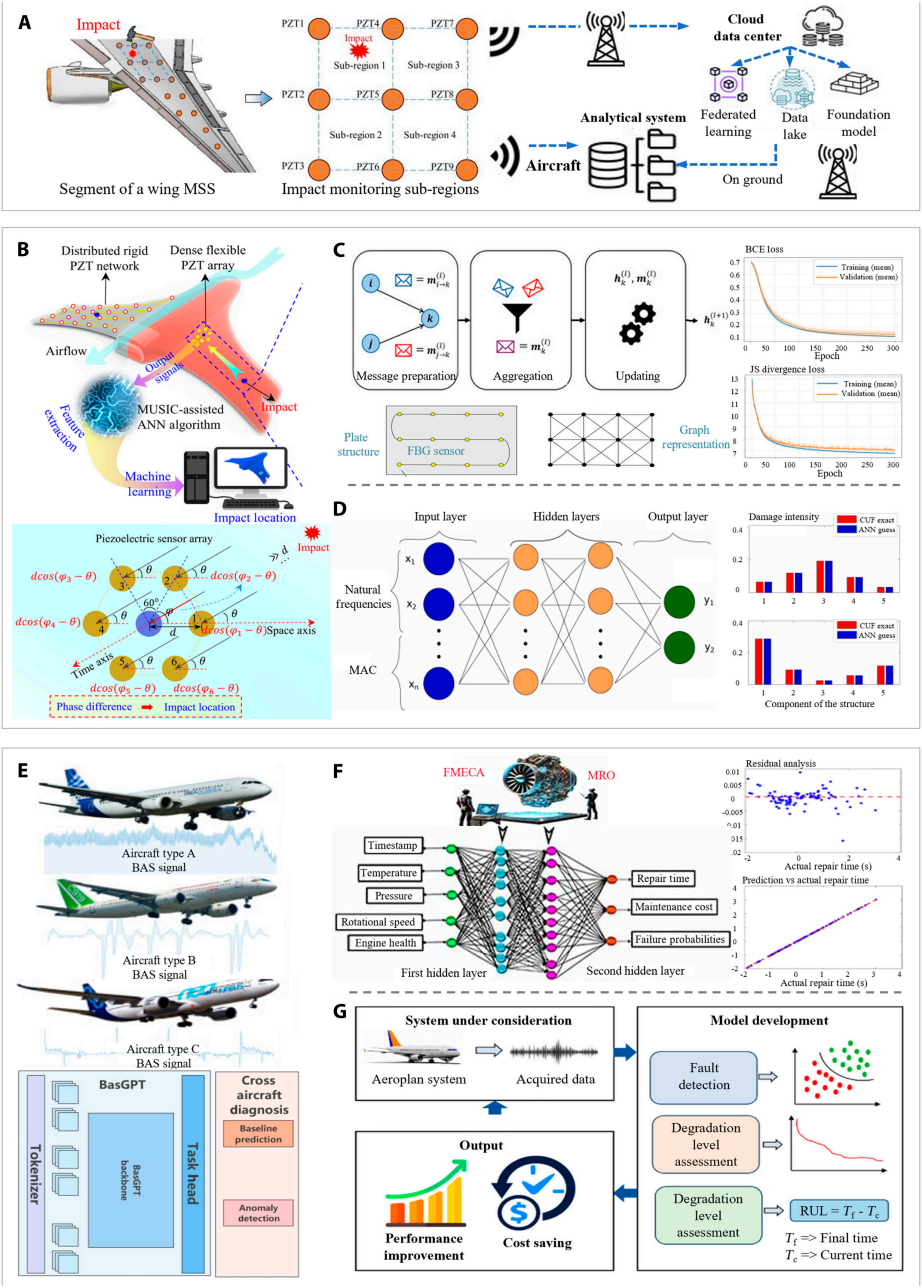
AI-empowered aircraft structural health detection. (A) Diagram of a lightning shield sensor. Health detection through on-board and cloud. (B) An SHM technology based on flexible miniaturized sensing array integration machine learning, combining miniaturized piezoelectric sensing array with backpropagation neural network (BPNN)-assisted multisignal classification (MUSIC) algorithm. Reproduced with permission from Ref. [104]. Copyright 2023, SAGE. (C) An aircraft damage monitoring and localization framework based on graph neural networks (GNNs); mean trend of training and validation loss for the classification and localization GNN. Reproduced with permission from Ref. [105]. Copyright 2023, The Author(s). (D) A damage detection method based on artificial neural networks (ANNs); comparison between exact the CUF solution (red bars) and the ANN output (blue bars) for a 3-stringer spar. Each graph represents a structure with all components damaged. Reproduced with permission from Ref. [107]. Copyright 2021, Elsevier. (E) BasGPT model design: A prompt token pool and CLS token pool for different aircraft types are prepended and appended to signal tokens in the Tokenizer to model inter-aircraft signal differences; an efficient self-adaptive transformer handles tokens of arbitrary length and channels to obtain high-level semantic representations, which are used for anomaly detection and baseline prediction. Reproduced with permission from Ref. [112]. Copyright 2025, Elsevier. (F) A hybrid predictive maintenance framework for training artificial neural networks with historical engine sensor data to effectively predict failure probability, maintenance duration, and cost. Reproduced with Ref. [114]. Copyright 2025, The Author(s). (G) The overall prognostics and health management (PHM) process is composed of PHM design, PHM development, and PHM decision, aiming to outline the tasks required to establish a comprehensive PHM framework that guides the entire life cycle of PHM efforts. Reproduced with permission from Ref. [119]. Copyright 2024, Springer Nature.

The essence of aircraft SHM is monitoring all damages that may affect structural integrity and service safety [101]. As mentioned in the previous section, sensors can only output raw physical signals such as strain and vibration, which cannot be directly mapped to core SHM requirements—including “whether the structure has damage, where the damage is located, and how severe the damage is”. Reliance on algorithms for signal interpretation and information processing is essential in the process from simple data collection to flight health assessment. Achieving the dual value of flight safety assurance and operation and maintenance (O&M) efficiency optimization is the core goal of aircraft SHM. Its implementation path is divided into 2 core directions: real-time damage state monitoring based on iFlexSense and systematic health management for the entire life cycle (Fig. 6A). These 2 directions form a collaborative closed loop of edge intelligence and cloud-based O&M, jointly constructing a full-chain health assurance system for aircraft.

Among them, real-time damage state monitoring takes the flexible intelligent skin as a carrier, capturing abnormal signals such as strain and vibration [102,103]. However, flexible sensors can only be deployed at limited discrete locations. For damage localization in uncovered areas, it is necessary to rely on algorithms to infer the global structural state from local sensing signals. This task falls under the supervised learning framework for classification tasks, focusing on rapid identification of equipment current states from distributed local sensing signals—addressed by lightweight node-based NNs. Zhu et al. [104] propose a large-area monitoring technology combining flexible micro piezoelectric sensor arrays and machine learning algorithms. It adopts the MUSIC-aided back propagation neural network (BPNN) algorithm to realize adaptive learning and prediction of impact location, and the accuracy rate of impact location reaches more than 90% (Fig. 6B). The MUSIC-aided BPNN is essentially a shallow feedforward NN based on MLP. It can efficiently establish the nonlinear mapping between multisensor inputs and state outputs, without relying on the complex automatic feature learning mechanism of deep NNs. The primary goal of the real-time health monitoring module is damage detection and localization. Del Priore and Lampani [105] designed 2 types of graph neural network (GNN) architectures that optimize feature processing logic for spatial structural damage localization, adapting to the spatial topological characteristics of aircraft structures (binary damage detection + spatial probability distribution localization), achieving a localization error of only 3% of the wingspan (Fig. 6C). Zheng et al. [106] used the improved Fisher Discriminative Dictionary Learning method, which can identify loads at 8 corrugated crests with only 2 FBG sensors, achieving 100% localization accuracy. After damage detection and localization, it is necessary to evaluate the severity of damage. Pagani et al. [107] proposed a damage detection method based on artificial neural networks (ANNs). Its core network employs a single-hidden-layer architecture to avoid redundant computations. Using modal parameters as inputs and leveraging the nonlinear mapping capability of MLPs, it efficiently establishes the mapping between feature inputs and structural damage location/severity, enabling accurate detection of damage in each structural component (Fig. 6D). It is worth noting that signals measured by sensors are the superposition of structural vibration, environmental interference, and operational noise. Among them, the amplitude of environmental interference and operational noise is often 10 to 100 times that of damage signals [108]. Thus, algorithms are essential for interference compensation and damage signal extraction. Furthermore, the power consumption in the airborne situation is always an important point. Wang et al. [109] have developed a miniature ultralow-power wireless multiparameter monitoring system suitable for aircraft smart skin, with an average power consumption of as low as 7.59 mW/m^2^ (1 to 3 orders of magnitude lower than traditional systems). In addition, spiking neural networks (SNNs), as the latest generation of AI, employ a unique event-driven mechanism based on the “wake-on-demand” principle rather than continuous monitoring. Specifically optimized for airborne ultralow-power consumption requirements [110], SNNs substantially lower the power consumption of flight systems, providing technology for the improvement of the endurance of mainstream aircraft and the landing of new energy aircraft.

Cloud-based intelligence compensates for a critical conflict in real-time damage monitoring—sacrificing computational scale for rapid response—providing a solution for scenarios requiring massive computing power such as long-term optimized O&M and life cycle management of aircraft [111]. Most life cycle health management tasks belong to regression tasks in supervised learning (e.g., remaining useful life [RUL] prediction), while semisupervised or unsupervised learning paradigms are adopted for extreme scenarios lacking prior data. Characterized by reliance on large-scale long-time series historical monitoring data, these tasks require network architectures with capabilities of long-term temporal dependency capture, deep feature mining, and global pattern fitting. Wang et al. [112] proposed a self-supervised learning foundation model (BasGPT). This model leverages deep feature extraction and multidimensional data fusion to perform full-life cycle diagnostics for cross-type bleed air systems. It addresses the challenges of data scarcity for new aircraft types (e.g., C919) and poor cross-type compatibility, enabling the transfer of diagnostic knowledge from mature aircraft types to new ones (Fig. 6E). Predicting the RUL of structural components using historical data is a critical module in long-term aircraft monitoring and predictive maintenance [113]. Leveraging large-scale server clusters and distributed computing architectures, flexible smart skins not only receive and transmit real-time data but also integrate historical flight parameters, maintenance records, and material fatigue test data. This breaks through the storage and computing power limitations of airborne embedded systems, avoiding problems such as low RUL prediction accuracy and simplistic O&M schemes caused by insufficient local computational scale. Dagal et al. [114] integrate ANN with failure mode and effects criticality analysis (FMECA). By combining the nonlinear fitting capability of ANNs and the systematic risk analysis framework of FMECA, they compensated for the poor interpretability of stand-alone NNs, while using engine sensor data (e.g., temperature, pressure, and vibration) to predict failure probability, maintenance duration, and costs (Fig. 6F). Li et al. [115] developed a probabilistic individual aircraft tracking model based on dynamic Bayesian networks, which captures the temporal evolution patterns of structural damage via probabilistic modeling. It is capable of predicting crack lengths over the next 10,000 cycles in the width of the 95% confidence interval, realizing crack evolution tracking and RUL prediction. Supported by the powerful computing power of the cloud, multitype aircraft data collaboration and algorithm iteration enable the construction of more universal and accurate health management models. For example, based on federated learning technology, while protecting the data privacy of each airline, damage data of smart skins from different aircraft types can be aggregated to optimize the generalization ability of RUL prediction algorithms [116]. This results in a 15% to 20% reduction in prediction errors compared to a single airborne model, while improving prediction stability by more than 25% under different operating conditions [117,118]. Raouf et al. [119] achieved landing gear fault diagnosis and RUL prediction based on multi-AI technologies, covering fault detection and isolation, RUL prediction, and maintenance decision-making, realizing full-life cycle health management (Fig. 6G). Basora et al. [120] proposed a semisupervised anomaly detection framework. Utilizing a semisupervised learning paradigm to extract implicit degradation features from unlabeled data, this method resolves the issue of anomaly identification in data-scarce scenarios. It addresses the key challenge of limited fault samples in the full-life cycle health monitoring of aircraft fleet cooling units, reducing the overall fleet maintenance cost by 15%. In addition, linking the health assessment results output by the model with the airline’s O&M system can generate globally optimal maintenance plans by integrating flight scheduling data, ensuring flight safety while minimizing O&M costs to the greatest extent [121]. A comparison of typical models for different detection tasks is presented in Table 5.

**Table 5.** Comparison of typical models for different detection tasks

	AI model	Targeted aerospace task	Core methodology	Computing hardware	Power consumption	References
Classification task	MUSIC-assisted BPNN	Shock monitoring and positioning	MUSIC spatial spectrum estimation + BPNN nonlinear mapping	Laptop-grade hardware	–	[104]
	Bi-architecture GNN	Structure damage localization	Global topological feature aggregation + GNN inference	Consumer-grade discrete graphics card (RTX 3050/4050 4 GB)	Total system <100 W	[105]
	ANN	On-board multiparameter monitoring	Vibration frequency extraction and recognition + ANN fitting	Intel i7-12700H/AMD R7-6800H	Full process ≤ 0.5 kWh	[107]
	Spiking CNN	Defect classification	ANN-SNN weight mapping + sparse pulse sequence learning	Neuromorphic hardware (IBM TrueNorth/Intel Loihi et al.)	Event-driven and low-power consumption	[110]
Regression task	Generative pretrained transformer	BAS baseline signal prediction	Cross-model signal tokenization + adaptive transformer modeling	NVIDIA RTX 3090/4090	Total system: 3,500 W	[112]
	Dynamic Bayesian network	Structural health monitoring of fatigue crack growth	Fusing signal spectrum estimation + NN fitting	Stand-alone workstation (Intel Xeon/AMD Threadripper)	–	[115]
	BPNN	Fault detection/isolation and remaining useful life prediction	Multidata fusion + Gaussian process regression prediction	Jetson Xavier NX/airborne embedded chip	<500 W	[119]
	Fully connected autoencoder	Anomaly detection in prognostics and health management	Semisupervised autoencoder + aviation time series anomaly detection	Workstation-grade GPU	1.5 kWh	[120]

### Aerodynamic field super-resolution reconstruction

**Fig. 7.**
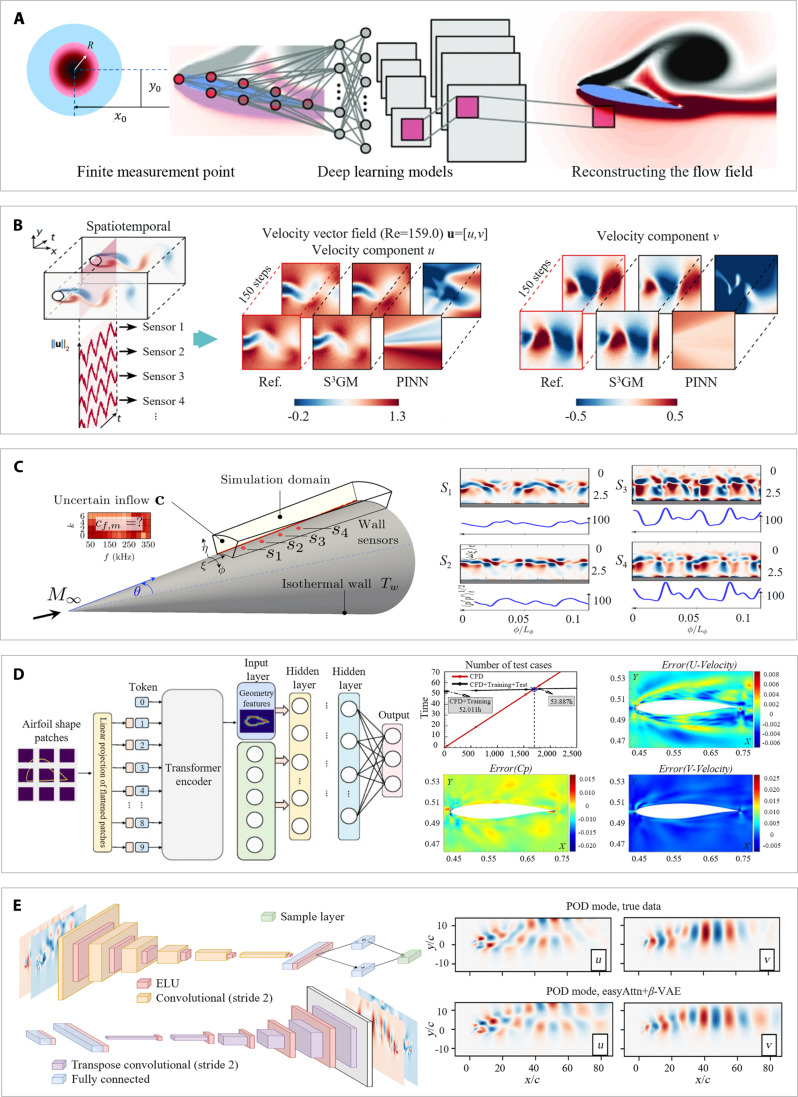
Flow field reconstruction and performance validation based on sparse measurement points: (A) Reconstruction of unsteady eddy current field based on surface sparse pressure measurement point. (B) Illustration of the measurements located at a downstream cross-section, and each of the measured signals is a long-time sequence representing the amplitude of velocity; a sample of the velocity vector fields measured by PIV (top, denoted as Ref.), predicted by S3GM (middle), and predicted by PINN (bottom). Reproduced with permission from Ref. [128]. Copyright 2024, Springer Nature. (C) The schematic of flow configuration for a slender cone at Mach 6; spanwise variation of time-averaged wall-pressure root mean square and streamwise vorticity at sensors 𝑠1 to 𝑠4. Reproduced with permission from Ref. [130]. Copyright 2022, Cambridge University Press. (D) Airfoil flow field prediction network architecture based on attention mechanism. The comparison of the time and the absolute error diagram between CFD calculation and DAN prediction. Reproduced with permission from Ref. [134]. Copyright 2023, AIP Publishing. (E) Model architecture and case schematic: β-VAE encoder and decoder. Analysis of predicted flow fields: Comparison of POD modes between the ground-truth and predicted flow fields, demonstrated for the case with Re = 100 and α = 80°. Reproduced with permission from Ref. [135]. Copyright 2024, The Author(s).

Aircraft operate in complex flow field environments, and the flow state of wing surrounding flow is a core factor determining aircraft flight safety and aerodynamic lift-drag characteristics. It is particularly crucial to accurately acquire the distribution of key physical parameters on the wing surface and the spatiotemporal evolution characteristics of the surrounding flow field. However, the flow field around the wing features highly nonlinear and strongly coupled complex evolution characteristics [122,123]. Constrained by the spatial resolution of existing measurement methods, full-scale, high-resolution direct observation is nearly unachievable in wing-related scenarios. Accordingly, the importance of flow field reconstruction technology has become increasingly prominent. This technology can recover global flow field information based on limited discrete measurement point data [124,125] (Fig. 7A), resolving the core contradiction between limited observation and full-field analysis requirements, and is an inevitable choice to connect experimental data, numerical simulation, and engineering applications.

The evolution of flow field reconstruction methods for wing surfaces is closely intertwined with the growing demand for “accuracy–efficiency” in aerospace engineering. Methods for reconstructing aircraft wing surface flow fields from sparse points are generally categorized into 2 types: data assimilation (DA) and data-driven methods. DA methods, centered on strong physical constraints, integrate sparse observations with aerodynamic control equations to ensure the physical consistency of flow field reconstruction [126]. They effectively avoid pronounced errors of early physics-unconstrained methods in high-gradient regions such as wing leading-edge shocks and trailing-edge separation vortices, thus becoming the core technical pathway for high-precision scenarios like airworthiness certification and hypersonic flight. These methods are based on aerodynamic control equations and integrate sparse observation data via DA techniques [127], with the Ensemble Kalman Filter and variational assimilation as typical representatives. Li et al. [128] proposed a sparse sensor-assisted fractional generative model that reliably reconstructs the spatiotemporal field in unobserved regions using only 1% sparse flow field data, even at high noise levels (Fig. 7B). To further balance the accuracy and efficiency of DA techniques, He and Liu [129] proposed the LSE (linear stochastic estimation) + continuous adjoint DA strategy, effectively expanding the application scenarios of DA. In addition, Buchta et al. [130] applied nonlinear variational DA at Mach 6 to estimate the unknown flow field over an elongated cone from discrete wall pressure measurements (Fig. 7C). Shu et al. [131] proposed a “Denoising Diffusion Probabilistic Model + Partial Differential Equation residual layer constraint” framework for high-fidelity data reconstruction, reinforcing DA’s core advantage in physical constraints. However, DA methods suffer from notable limitations: as they require solving large-scale linear or nonlinear equations derived from control equations, calculating a single flow field typically takes hours or even days, resulting in a substantial mismatch with scenarios demanding high real-time response speeds.

Data-driven approaches, with NNs as their core, can be mainly categorized into 2 types: pure data-driven models and physics-informed learning frameworks. When applied to flow field reconstruction, physics-informed neural networks (PINNs) exhibit distinct characteristics and performance compared with pure data-driven networks, and their respective advantages and limitations are complementary. PINNs exhibit superior generalization ability and robustness, with strong resistance to noisy data, while inherently featuring physical consistency and interpretability. Nevertheless, their fitting accuracy deteriorates notably in reconstructing flow fields with high Reynolds numbers and strong nonlinear turbulence. Especially in large spatiotemporal domain flow field reconstruction, the training procedure becomes complicated and computationally time-consuming. By constructing lightweight models or introducing a small number of physical priors, these methods learn the mapping between sparse measurement points and high-resolution flow fields and accomplish reconstruction in a matter of seconds. They substantially enhance the efficiency and robustness of flow field reconstruction, rendering this approach one of the most extensively applied technical directions at present [132,133]. The primary advantage of pure data-driven models lies in high-resolution reconstruction of complex turbulent flows. Trained on large-scale labeled datasets, these models can accurately capture the complex nonlinear characteristics and fine-scale structures of flow fields. Without solving intricate physical constraint equations, they enable high-resolution flow field reconstruction within seconds. Nevertheless, such models rely heavily on large-scale labeled data with high spatiotemporal resolution. Their reconstruction accuracy degrades drastically under sparse or noisy measurements, often leading to missing flow details and distorted structures. Generalization is constrained by the distribution of training data, with substantial performance degradation when flow conditions exceed the training range or the sensor layout is modified. Lacking inherent physical constraints, these models exhibit poor interpretability. Zuo et al. [134] achieved end-to-end flow field reconstruction based on the Transform encoder’s deep attention network (DAN). Its batch prediction takes only 23 s (115 s for a single CFD case), greatly reducing the iteration period of aerodynamic design (Fig. 7D). For the problem of high-dimensional and nonlinear characteristics of complex fluid flow fields such as turbulence, Solera-Rico et al. [135] propose an end-to-end AI framework of “β-VAE extraction of spatial features + transformer prediction of temporal dynamics”, which greatly reduces the computational complexity and adapts to the rapid simulation and prediction requirements of engineering scenarios (Fig. 7E). Additionally, the spatiotemporal super-resolution method based on supervised machine learning proposed by Fukami et al. [136] improves efficiency by 3 orders of magnitude compared with DNS (which takes several hours). Different deep learning models have been applied to flow field-related tasks: multiscale temporal-path UNet (MST-UNet) enhances spatiotemporal flow field accuracy [137], the unsupervised deep learning model based on CycleGAN enables super-resolution reconstruction of turbulent flow fields without unpaired data [138], and the combination of GNN and MST pruning optimizes hypersonic flow field reconstruction efficiency with only minimal physical priors [139]. In recent years, the integration of NNs with other technologies has further broken through performance bottlenecks. Zhao et al. [49] proposed a PMNN (POD + MLP) model, which can reconstruct the flow field at the same resolution as that of full CFD simulations (equivalent to 222 grid points) with only 5 measurement points, and the entire process takes merely 2 ms. To address the issues of sparse flow field data or regional data missing, Xu et al. [140] use the Navier–Stokes equations as a loss constraint function. Even when the data sparsity reaches 1% or the data in the core wake region is truncated, the proposed model can still quickly and accurately reconstruct the wake field.

In the future, it can be anticipated that with the in-depth coupling of physical prior information and data-driven models, the physical consistency of NN-based methods will be further enhanced, and the proportion of integrated applications of the 2 types of methods (NN-based and DA-based) will continue to increase. Together, they will promote the development of aircraft flow field reconstruction technology using sparse measurement points toward real-time, intelligent, and highly reliable directions.

## AI-Enhanced Adaptive Actuating

The technical merit of iFlexSense extends far beyond its capability for perception–decision information processing. The technical merit of iFlexSense extends far beyond its capability for perception–decision information processing. As the core module for AI- enabled UAVs, it constructs a bridge between in situ flight-related data acquired via structural skin sensing (e.g., pressure, shear stress, and strain) and the signal reconstruction and situational awareness of onboard AI, thereby enabling the directed translation and synergistic coupling of raw sensory inputs into UAV-specific decision outputs. By further advancing its active actuation functionalities, iFlexSense forms an integrated closed-loop intelligent system characterized by perception–actuation–control synergy. On the one hand, leveraging morphing technology to realize active actuation such as dynamic regulation of wing configurations, and combining preacquired sensing data with AI decision-making, it accomplishes tasks like real-time optimization of aerodynamic characteristics. On the other hand, the microstructures themselves can directly endow the skin with specific functional properties—for instance, hydrophobic microstructures and electromagnetic metasurface—enabling the fulfillment of key application requirements without additional complex actuation units. In this way, the intelligent skin evolves from a mere information-sensing system into an intelligent microsystem with active regulation capabilities.

### Active anti-icing and deicing

**Fig. 8.**
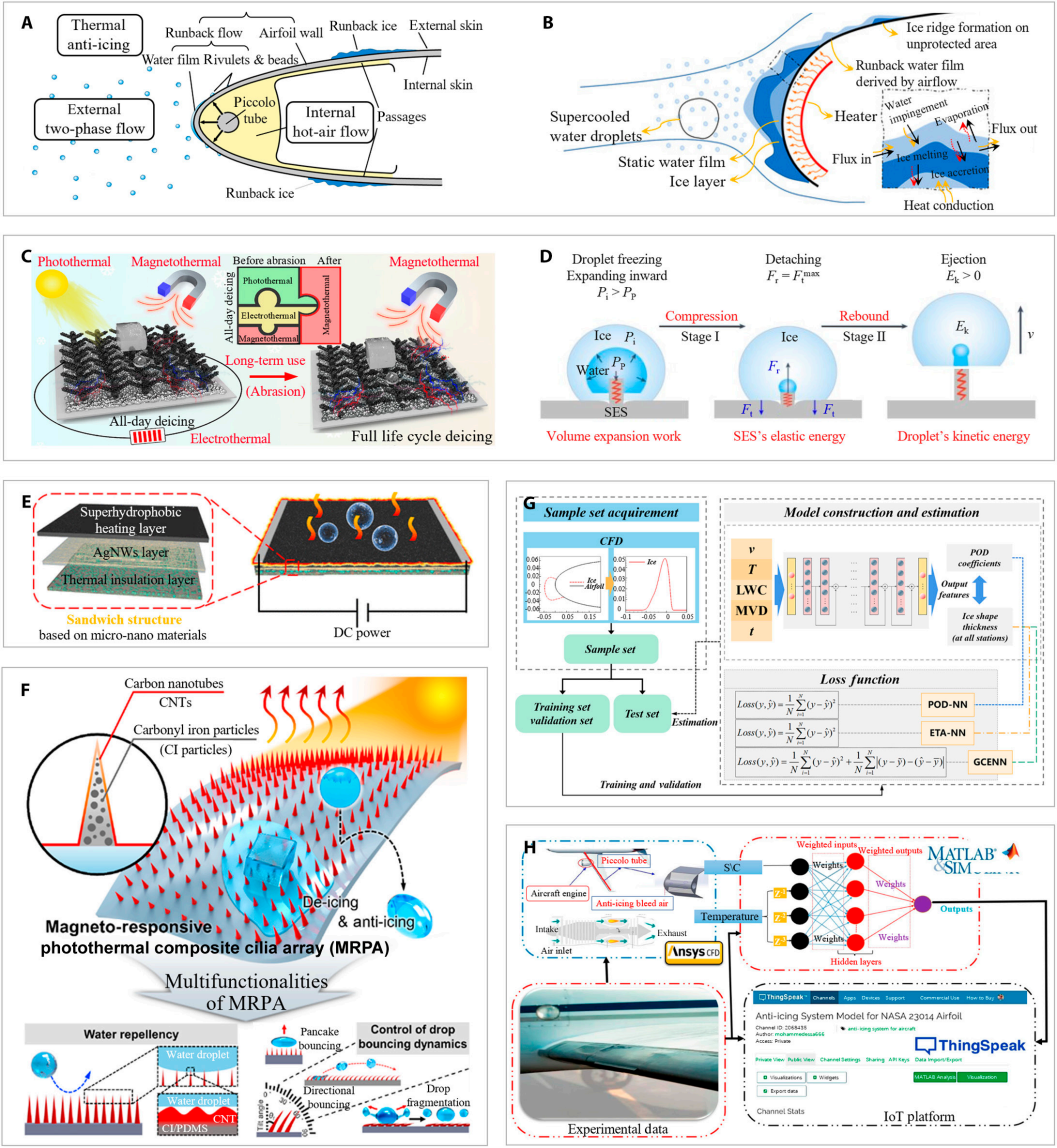
AI-empowered aircraft deicing and icing prediction (A) Schematic of bleed-air anti-ice system divided into 3 regions: the external 2-phase flow, the hot-air internal flow, and the airfoil wall. Reproduced with permission from Ref. [147]. Copyright 2010, The Author(s). (B) Schematic illustration of the electrothermal deicing process in aircraft in-flight icing applications, including mass exchange, sensible heat exchange, and latent heat exchange. Reproduced with permission from Ref. [148]. Copyright 2022, Elsevier. (C) Schematic illustration of a novel superhydrophobic graphene@NiO/Ni surface and the use for anti-icing, photo-/electro-/magnetothermal deicing, and full life cycle deicing. Reproduced with permission from Ref. [151]. Copyright 2025, Wiley-VCH GmbH. (D) Structured elastic surface with spring-like pillars and wettability contrast: droplet ejection driven by 2-stage energy conversion for surface deicing. Reproduced with permission from Ref. [152]. Copyright 2024, Springer Nature. (E) An electrothermal/superhydrophobic coating featuring a sandwich structure was proposed for anti-deicing applications. Reproduced with permission from Ref. [153]. Copyright 2024, American Chemical Society. (F) A multifunctional magneto-responsive photothermal composite cilia array (MRPA) with excellent anti-icing/deicing performance is demonstrated, which can inhibit, delay, and remove ice on both supercooled impinging and stationary droplets. Reproduced with permission from Ref. [154]. Copyright 2022, Elsevier. (G) Frame diagram of airfoil icing prediction modeling based on neural networks. Reproduced with permission from Ref. [158]. Copyright 2024, Emerald Publishing. (H) An intelligent ANN model is developed based on machine learning (ML) and the Internet of Things (IoT). It predicts the thermal performance characteristics of a partial-span wing anti-icing system using experimental and CFD data. Reproduced with permission from Ref. [159]. Copyright 2023, The Author(s).

Aircraft icing is a long-standing severe challenge in the aviation field. When an aircraft flies in environments such as clouds and supercooled water mist, supercooled water droplets will rapidly condense and freeze on parts including the fuselage, wings, and engine air inlets [141]. Icing can seriously damage the aerodynamic configuration of an aircraft, leading to lift loss and increased drag within a very short period of time, and may even result in catastrophic incidents such as stall that could cause casualties [142]. Aircraft anti-icing and deicing can be divided into anti-icing systems and deicing systems. Anti-icing technology prevents ice formation or adhesion on aircraft surfaces through nonactive energy input [143]. Among various anti-icing technologies, superhydrophobic surfaces have attracted extensive attention due to their excellent ice-retarding performance, icephobicity, as well as no energy consumption and low cost [144]. Their working principles can be roughly categorized into 2 types: utilizing microstructural design [145] and leveraging inherent material properties [146]. However, superhydrophobic surfaces can only inhibit icing to a limited extent rather than completely prevent it; when the ice layer accumulates to a certain thickness, deicing systems take effect. The traditional deicing method involves the use of hot air and an electric heating system [147,148] (Fig. 8A and B). The hot air system relies on the engine air intake or independent heater to provide heat energy, which requires a large amount of fuel to be converted into heat energy, and there are multistage losses in the energy conversion process, and most of the energy is lost in the pipeline transmission and heat exchange process; the electric heating system starts up with high instantaneous power, which puts great pressure on the aircraft power supply system. In addition, the weight of the whole set of equipment usually reaches hundreds of kilograms, directly increasing the load of the aircraft.

Flexible intelligent skin provides a new possibility for aircraft anti-icing and deicing. The flexible smart skin has achieved considerable weight reduction in structure, reducing the weight by more than 60% compared with traditional electric heating systems [149], while reducing energy consumption by about 50% [150]; during maintenance, only faulty modules need to be replaced, which considerably reduces operating costs. It can be fitted on any complex curved surface to achieve full coverage, avoiding the risk of local icing resulting from uneven heating on the fuselage surface in traditional deicing methods, and can further integrate both an ice condition sensing unit and an anti-icing/deicing module. Xu et al. [151] prepared a superhydrophobic graphene @NiO/Ni surface with triple heat conversion capability. The internal nickel layer imparts magnetocaloric deicing capability, which can achieve high-efficiency deicing throughout the life cycle (Fig. 8C). Zhang et al. [152] reported a structured elastic surface integrated with spring-like columns. These columns can store the work from volume expansion of frozen droplets as elastic energy, then release it rapidly as kinetic energy within milliseconds to achieve spontaneous ejection of the droplets (Fig. 8D). In recent years, active/passive coupled deicing technology has exhibited greater adaptability and effectiveness in different scenarios. Li et al. [153] show a sandwich-structured composite coating integrating both electric heating and superhydrophobic functions: its top layer is composed of hydrophobically modified epoxy resin and carbon black, while the silver nanowires (AgNWs) in the middle layer are used to adjust resistance and enhance electrothermal performance (Fig. 8E). Lee et al. [154] present a multifunctional magnetically responsive photothermal composite cilia array (MRPA). The MRPA exhibits immediate and reversible structural bending motion while maintaining superhydrophobic properties, enabling effective prevention, delay, and removal of ice accumulation (Fig. 8F).

AI-enabled flexible intelligent skin enables intelligent predictive decision-making and active deicing. Via integrated multiparameter sensors and combined with flight conditions, it establishes an “icing risk prediction model” to activate low-power preheating before supercooled water droplets freeze, realizing the mode of “anti-icing first, deicing supplementary”. Jingxin et al. [155] tested an integral glass fiber composite airfoil with superhydrophobic coating and embedded electrothermal film (SHS-EET). Icing wind tunnel experiments showed that SHS-EET reduced ice shedding cycle by 64.6% and energy consumption by 72.3%. AI can also predict icing location and extent; leveraging real-time flight parameters from sensors, it dynamically identifies key icing characteristics such as location, thickness, and morphology. Compared with traditional machine learning methods, identification accuracy in complex icing scenarios improved by ~15% to 20% [156]. Løw-Hansen et al. [157] embedded electrothermal elements in UAV wings, monitoring ice shedding in real time via Kalman filter and continuous wavelet transform. AI algorithms predicted ice peeling time based on temperature changes and dynamically adjusted heating strategies to reduce energy consumption. Suo et al. [158] proposed a wing icing prediction model based on geometric constraint-enhanced NN. It enables rapid airfoil icing prediction (6 ms per case) and exhibits good generalization performance (Fig. 8G). Abdelghany et al. [159] adopted the Internet of Things (IoT) integrated with an ANN to predict the thermal performance of anti-icing systems. The proposed model is calibrated with experimental data to further optimize the heating power distribution, thereby improving the overall deicing efficiency (Fig. 8H). With “flexibility”, flexible intelligent skin breaks through structural adaptability limitations of traditional methods; with “intelligence”, it upgrades from “single deicing” to “multifunction integration and predictive regulation”, becoming one of the core development directions in future aircraft anti-icing and deicing.

### Metamaterial surface for electromagnetic stealth

**Fig. 9.**
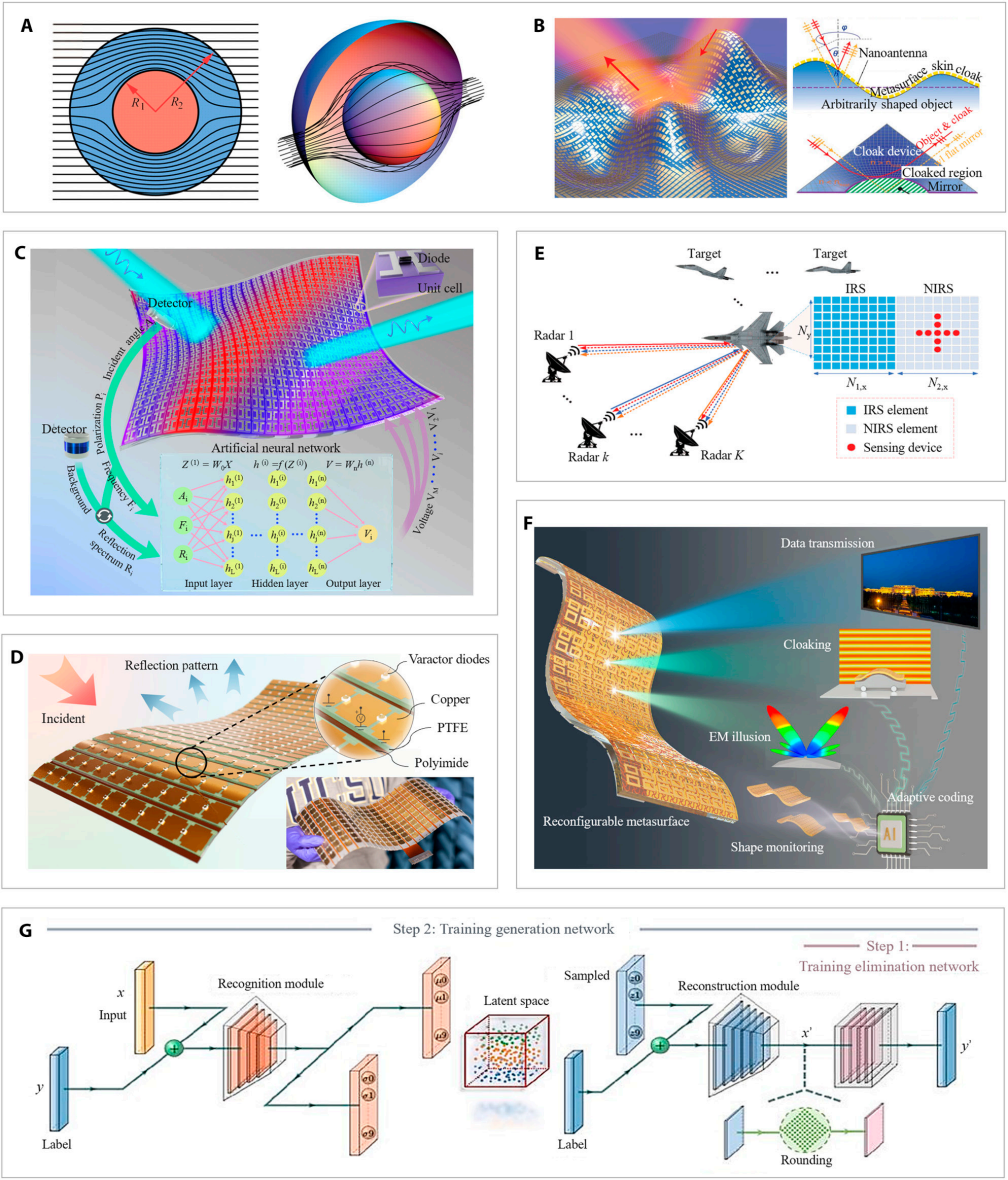
AI-empowered aircraft electromagnetic stealth (A) A 2-dimensional (2D) cross-section of rays striking the system and a 3D view of the same process (the electromagnetic wave still propagates in the original direction after passing through the designed anisotropic cloaking sphere). Reproduced with permission from Ref. [164]. Copyright 2006, The Author(s). (B) A 3D illustration of a metasurface skin cloak. The skin cloak is an ultrathin layer of nanoantennas (gold blocks) covering the arbitrarily shaped object; schematic view of a cross-section of the metasurface skin cloak and a conventional carpet cloak. Reproduced with permission from Ref. [165]. Copyright 2015, The Author(s). (C) Schematic of a deep-learning-enabled self-adaptive metasurface cloak. Detected background information is converted to the target reflection spectrum for each meta-atom via the wave reconstruction method and fed as input to the pretrained ANN. Reproduced with permission from Ref. [169]. Copyright 2020, Springer Nature. (D) Illustration of the conformable rigid-flex printed circuit design. The substrate of each column is made with 1.57-mm semirigid reinforced PTFE material designed for microwave applications. The small panel in the bottom right corner shows the prototype photo. Reproduced with permission from Ref. [173]. Copyright 2023, The Author(s). (E) Moving targets equipped with IRS-aided electromagnetic stealth against multiple mono-static radars. Reproduced with permission from Ref. [178]. Copyright 2024, IEEE. (F) Schematic illustration of the flexible intelligent surface platform (FISP). Transient shape information is fed to the BSM driven by ANN to adaptively determine the bias voltage for metasurface reconfiguration, which achieves the robustness of FISP to dynamic deformations. Reproduced with permission from Ref. [181]. Copyright 2025, The Author(s). (G) Architecture of stochastic-evolution learning that drives the autonomous invisible drone. The proposed network consists of 2 cascaded networks, namely, the generation network and the elimination network. Reproduced with permission from Ref. [182]. Copyright 2024, The Author(s).

Electromagnetic stealth is a crucial capability for aircraft to survive and operate in the modern complex electromagnetic countermeasure environment, which is directly related to the aircraft’s own safety and the success or failure of missions. Enemy electromagnetic detection devices such as radars can locate aircraft by emitting electromagnetic waves and receiving the reflected signals from targets [160]. There are numerous historical cases of aircraft damage caused by electromagnetic exposure. Initially, stealth technology was based on surface coatings for passive absorption/reflection, and later, metamaterials provide new possibilities for stealth technology by actively regulating the propagation path of electromagnetic waves [161]. The metamaterial stealth relied on complex 3D structures (thickness reaches centimeter level), which cannot meet the requirements of “lightweight and thin” in the aerospace field [162]. To address the limitations of 3D metamaterials, researchers compressed the meta-structure from a “3D bulk” to a “2D interface”—the thickness of metasurface is usually less than 1/20 of the wavelength [163]. Its main working mechanisms are divided into 2 types: light diffraction-based stealth and scattering cancellation-based stealth [164,165] (Fig. 9A and B). Integrating electromagnetic metasurface into the flexible intelligent skin of aircraft enables electromagnetic wave scattering reconstruction or phase modulation through the subwavelength microstructures on the metasurface, making this integrated system a new stealth paradigm based on wavefront engineering [166,167].

The involvement of AI technology provides core support for the dynamic stealth capability of aircraft flexible intelligent skins. Its value is concentrated on optimizing the real-time performance and precision of metasurface regulation. The system can dynamically adjust the bias voltage strategy of metasurface elements based on real-time analysis of parameters of incident electromagnetic waves such as reinforcement learning algorithms. This reduces the reflectivity regulation response time from milliseconds to even nanoseconds and expands the broadband stealth bandwidth by over 30% [168]. Qian et al. [169] took the lead internationally in developing a new generation of intelligent stealth devices based on deep learning. The devices can dynamically adapt and integrate with the characteristics of the background electromagnetic environment in only 15 ms (Fig. 9C). However, the experimental object is a perfect electrical conductor bump of a specific size, and the stealth effect can only be achieved for electromagnetic waves in a specific vibration direction. As research continues, stealth devices begin to develop into universal adaptations and achieve full polarization regulation (capable of responding to electromagnetic waves in all vibration directions) [170,171]. The active frequency selective surface (FSS) structure controlled by PIN diodes proposed by Cerveny et al. [172] can achieve full polarization reflectivity regulation in the 9 to 13 GHz band, but it is still difficult to solve the problem of multiband interference in complex electromagnetic environments. In recent years, NNs have been increasingly applied in complex electromagnetic/photonic systems. The pure NN-driven solution does not involve an iterative process during the prediction phase, which is crucial for on-site systems that require rapid responses. This characteristic has facilitated the development of an emerging concept—intelligent metasurface [173] (Fig. 9D). This intelligent metasurface can cancel the interference signal through NN in the electromagnetic environment where other scatterers exist, and without retraining, it can integrate the automatic regulation strategy of environmental sensing information, responding to design targets of multiple reflection modes with extremely low on-site computing capacity and time [174]. Next-generation stealth systems impose more stringent requirements on secure electromagnetic transmission, which demands reliable information delivery while avoiding electromagnetic radiation exposure. The radiation–stealth integrated metasurface provides a viable technical solution [175], enabling simultaneous high-efficiency dynamic radiation and low-observable full-polarization stealth within the same operating frequency band.

In addition to the exploration of general metasurface technology, research targeting radar detection, a typical application scenario, has also achieved new breakthroughs [176,177]. The core of radar detection is to achieve the maximum signal-to-noise ratio to improve the detection probability. The multiradar networking system proposed by Zheng et al. [178] has advanced metasurface technology from theoretical feasibility to engineering practicality (Fig. 9E). In addition, when an aircraft encounters wing deformation caused by airflow disturbance, it is also important to predict the attenuation trend of stealth performance in advance [179]. Xu et al. [180] proposed an intelligent reflecting surface that integrates integrated radiation and scattering control and integrated sensing and communication technologies. By conducting cross-dimensional fusion training on the sensed flow field pressure distribution data and metasurface electromagnetic scattering characteristic data, it can accurately modulate electromagnetic waves in real time according to dynamic variations of the external environment, thereby controlling the fluctuation of radar cross-section and achieving adaptive active stealth. Li et al. [181] demonstrated a flexible intelligent surface platform (FISP). This platform takes the metasurface’s own shape information as the core input, and the ANN-driven algorithm generates coding sequences by feeding data from the sensor array, with a response time of approximately 16.7 ms (Fig. 9F). Stochastic evolutionary learning (SEL)—a 2-stage mechanism that generates candidate solutions and selects optimal solutions—constitutes a core breakthrough at the algorithmic level (Fig. 9G). Qian et al. [182] innovatively adopted spatiotemporal modulation metasurface, breaking through the limitation that pure spatial-domain functional modulation can only be achieved by adjusting the spatial distribution or phase of metasurface elements, and completed outdoor flight tests across air–sea–land scenarios. This breakthrough is not only an upgrade of metasurface technology but also opens up a new paradigm of “flight autonomous stealth”, laying a foundation for the next generation of intelligent stealth equipment.

In the future, flexible intelligent skins in the electromagnetic stealth field must address core challenges such as high-density integration of metamaterials with skins and extreme environment adaptability, continue to advance toward multiband compatibility and AI-driven real-time adaptive electromagnetic regulation, and achieve dynamic stealth and synergistic adaptation with the environment.

### Active lift enhancement and drag reduction

**Fig. 10.**
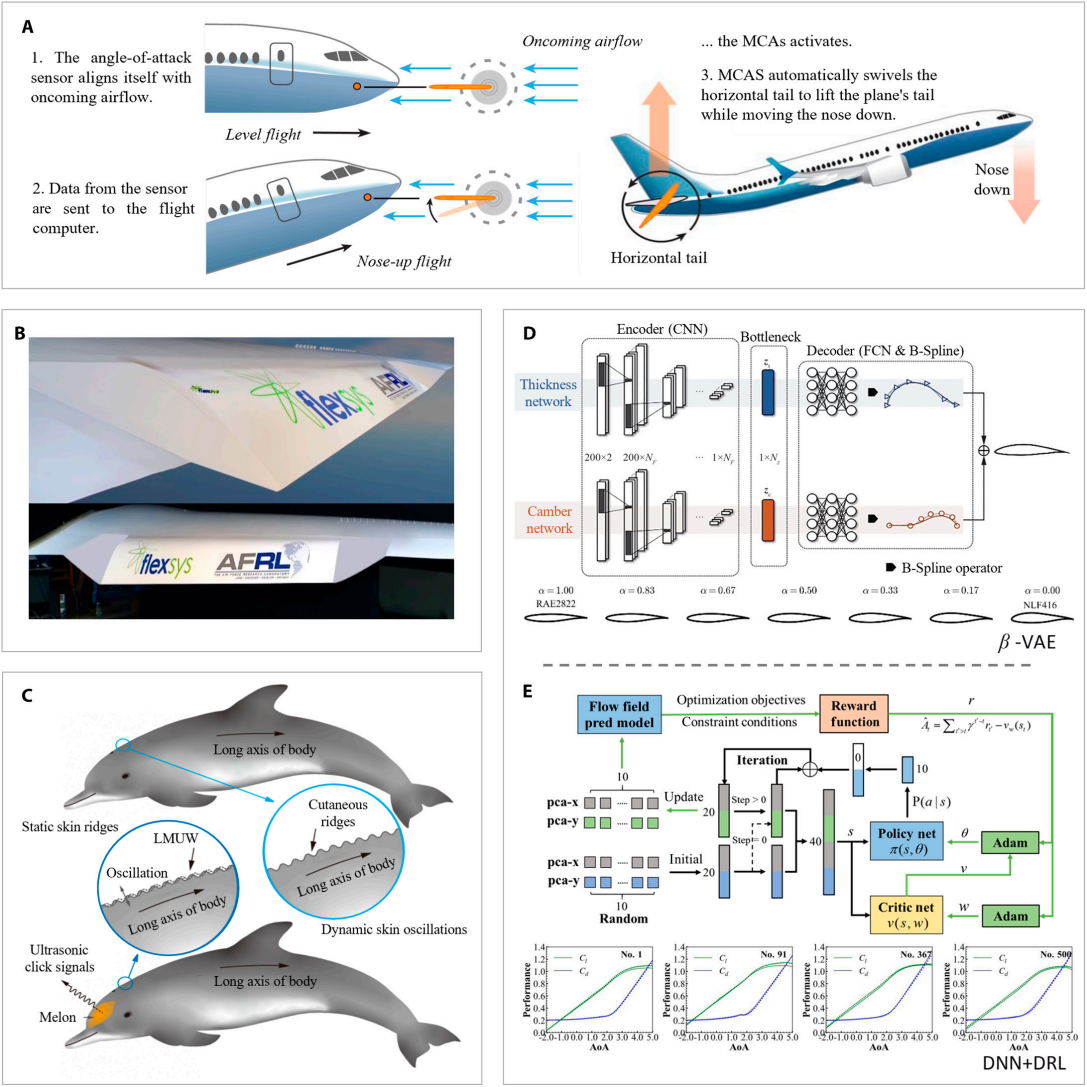
AI-empowered aircraft lift and drag reduction. (A) The principle of the Maneuvering Characteristics Augmentation System (MCAS) prevents aircraft from stalling. Reproduced with permission from Ref. [184]. Copyright 2020, The Author(s). (B) High-lift flaps of a Gulfstream III jet (NASA–Air Force test aircraft) were replaced with 19-ft multifunctional variable control surfaces on each wing. Reproduced with permission from Ref. [185]. Copyright 2018, Springer Nature. (C) Schematic of surface morphology on dolphin skin. Static cutaneous ridges perpendicular to the long axis of the dolphin’s body and dynamic skin oscillations on dolphin skin, in the form of LMUWs. Reproduced with permission from Ref. [199]. Copyright 2025, The Author(s). (D) Variational autoencoder-based generative modeling for independent control of airfoil thickness and camber distribution, enabling smooth geometry generation and full elimination of self-intersection, and airfoil geometries generated along the linear trajectory in the design space of VAE (*β* = 1.0 × 10^−8^). Reproduced with permission from Ref. [195]. Copyright 2024, American Institute of Aeronautics and Astronautics. (E) An intelligent optimization experience-learning framework combining a deep neural network (DNN)-based flow-field prediction model with a deep reinforcement learning (DRL)-based optimization method; variation of optimized lift and drag coefficients with angle of attack. Reproduced with permission from Ref. [196]. Copyright 2025, Elsevier.

The development of aircraft lift-enhancing and drag-reducing technology has always been confronted with the contradiction of balancing functional requirements and aerodynamic performance. Existing technologies often manifest themselves as “passive remediation” [183]. Taking the Boeing 737 MAX series as an example, to accommodate the LEAP-1B engines—with higher thrust and better fuel efficiency—the design team had to reposition the engines rearward and increase their size. This modification undermined the aircraft’s original pitch stability: under critical operating conditions such as low speed and high angle of attack, the aircraft nose tended to pitch up excessively, and there was even a risk of stall. To address this issue, Boeing developed the Maneuvering Characteristics Augmentation System (MCAS) [184]. Achieve “remedial control” by automatically pressing down the machine head under specific conditions (Fig. 10A). The shape of a traditional aircraft is fixed after the aircraft leaves the factory; it can only adapt to limited operating conditions through segmented adjustments of mechanical components such as flaps and slats. This highlights the bottleneck of current lift-increasing and drag-reducing technologies. The flexible smart skins can sense the airflow state in real time and dynamically adjust their curvature of the skin (macroscopic level) or local convex–concave structures (microscale surface), thus actively reducing drag and enhancing lift [185].

The flexible skins can achieve continuous bending of the wing along its full span and chord (Fig. 10B), and under AI enablement, the optimal airfoil profile for different operating conditions can be calculated to realize full-range airflow adaptation [186,187]. In addition, inspired by highly efficient aerodynamic organisms in nature, the flexible skin can achieve aerodynamic optimization by guiding airflow from surface protrusions and inhibiting separation from local oscillations [188,189]. The aerodynamic performance of the shark-skin-mimicking microgrooves is theoretically underpinned by the Reynolds-Averaged Navier–Stokes equations for incompressible turbulent boundary layers. Mimicking the microgroove structure of the shark skin—micrometer-scale rhombus-shaped protrusions and grooves—these microstructures constrain the spanwise near-wall flow, inhibit the development of streamwise vortices and hairpin vortices, and reduce turbulent Reynolds stresses, thereby stabilizing the near-wall flow into a low-fluctuation laminar-like state; the drag-reduction effect is optimal within the Reynolds number range corresponding to the laminar and laminar-turbulent transition regimes. The flexible skin uses the microstructure of protrusions and grooves to avoid airflow disturbance caused by lateral disturbances, delaying the transition of airflow from laminar to turbulent flow, thereby reducing frictional resistance [190], whereas the microstructure loses effectiveness or fails when the Reynolds number exceeds the upper bound of the optimal range (resulting in fully developed turbulence) or under high free-stream turbulence intensity and strong sudden disturbances, as intense turbulent mixing or an inherently unstable boundary layer negates the flow-guiding and disturbance-suppression effects. Furthermore, borrowing from dolphins to correct eddy and surface airflow disturbances in real time through microvibrations (Fig. 10C), by causing active disturbances such as micro-oscillations in specific areas of the skin, the external airflow is “sucked” into the separation zone and reattached to the wing surface, thereby suppressing the risk of stall [191].

The self-sensing capability and structural control function of flexible intelligent skins lay the foundation for aerodynamic optimization, while AI realizes the “perception–deformation–optimization” closed loop, enabling the system to evolve into active intelligent control. To address the challenge of limited aerodynamic datasets, it is crucial to enhance the model’s ability and efficiency in predicting aerodynamic parameters under unknown conditions. Studies such as cross-attention models incorporating physical information [192], denoising diffusion aerodynamic models [193], and generative design models based on conditional VAEs [194], among others, have been successively proposed. In the exploration of such generative models, the VAE framework, leveraging its advantage of flexible parameter regulation, has gradually been applied in airfoil design. Kang et al. [195] proposed a β-VAE-based airfoil parameterization method that can independently adjust physical parameters such as thickness and camber to generate smooth, nonself-intersecting airfoils (Fig. 10D). To meet more complex aerodynamic engineering requirements, Liu et al. [196] proposed a dual-module collaborative model: a deep neural network (DNN) rapidly outputting accurate flow field distributions; the deep reinforcement learning (DRL) optimization module continuously adjusts airfoil parameters via interactive learning with the DNN-predicted flow field, ultimately producing adaptive optimization strategies (Fig. 10E). Although there are relatively few studies in the literature on using flexible intelligent skins to actively alter surface protrusions or local oscillation states to intervene in airflow attachment and separation on wing surfaces, numerous studies have confirmed the drag reduction effect of transverse grooves [197,198]. Inspired by dolphin skin—whose dynamic microscale oscillations substantially affect the boundary layer near the skin—Wang and Liu’s research [199] demonstrated that this mechanism not only enables effective drag reduction through dynamic oscillations but also generates additional thrust.

It is foreseeable that morphing wings capable of intelligent adaptive deformation and real-time adaptation to multiple flight conditions will spearhead the development of future aircraft; meanwhile, research on the surface microstructures of flexible skins based on bionic design will also emerge as a core focus for advancing the aerodynamic performance of aircraft.

## Conclusion and Further Work

This review outlines the technical characteristics and development evolution of iFlexSense, and elaborates on its technical pathway to achieve full-scenario perception–decision–actuation by integrating multidimensional sensing/actuation units on ultrathin substrates and combining AI-enabled super-resolution field reconstruction algorithms. It conducts an in-depth analysis of the technology’s breakthrough applications in 3 core scenarios—anti-icing/deicing, electromagnetic stealth, and lift enhancement/drag reduction—as well as its substantial improvements to aircraft safety, survivability, and energy efficiency. This technology thoroughly breaks the inherent design limitations of traditional aircraft that passively adapt to aerodynamic environments, establishing a new paradigm featuring active airflow perception and dynamic adjustment of surface properties. Nevertheless, there remains room for further development and refinement of iFlexSense, and the following aspects are poised to become its core future development directions.

### Optimization of multimodal sensor units at the unit cell level

Unit cell modularization and high-density integration are the core future development directions of iFlexSense. Leveraging electronic row–column scanning and signal demodulation, multiphysical sensing modules are integrated into a single sensor unit cell, enabling synchronous acquisition and accurate resolution of multidimensional physical parameters at the same spatial position. This greatly improves the sensing system’s integration density and data integrity, while reducing redundant sensor deployment. However, the technology still needs further optimization in terms of large-scale mass production processes and cost control. Key breakthroughs are required in material costs and the stability of batch preparation, so as to promote its transformation from laboratory prototypes to large-scale commercial products.

### In-depth fusion of AI and edge computing

Edge intelligence represents a major trend in future development. Moving forward, focus should be placed on the lightweight development of AI algorithms: computational complexity can be reduced through techniques such as model compression and quantization, while deployment of edge computing modules enables the migration of data processing to the local skin system. This will substantially enhance the real-time performance of data processing and the efficiency of autonomous decision-making. Practical engineering application needs to address the power consumption constraint of edge hardware under aircraft limited energy supply, and the compatibility issue between lightweight models and dynamic physical signal noise in complex flight environments.

### Collaborative innovation of multifunctional drive technologies

In existing flexible intelligent skins, functions such as anti-icing/deicing, electromagnetic stealth, and lift enhancement/drag reduction are mostly designed independently. It is necessary to deepen the multifunctional collaborative design—for instance, integrating the electrothermal elements for anti-icing/deicing with the conductive network of stealth coatings in an integrated manner. This will effectively reduce system complexity and overall aircraft energy consumption, meeting the development requirements of next-generation energy-saving and multimission aircraft. Key engineering challenges include material interface peeling between integrated functional layers under cyclic mechanical stress (e.g., aircraft takeoff/landing), and uneven energy distribution in electrothermal–stealth integrated structures affecting both function efficiencies.

### Autonomous collaborative upgrading of embodied intelligence

Embodied intelligence, as the latest research direction of iFlexSense, has demonstrated its core potential. Going forward, efforts should focus on advancing the evolution from the basic perception–decision–actuation closed loop to advanced autonomous learning, dynamic environmental adaptation, and multitask collaborative decision-making. This will fully unleash the core value of iFlexSense as an embodied intelligent agent for next-generation aircraft, and meet the higher-level requirements for autonomous and collaborative development of next-generation intelligent aircraft in complex mission scenarios. From a practical implementation perspective, the real-time response lag of autonomous learning algorithms under high-dynamics flight conditions and the difficulty in validating multitask collaborative decision robustness across extreme weather and mission switching scenarios need to be resolved.

## Data Availability

The data that support the findings of this study are available from the corresponding author upon reasonable request.
